# Cytosolic copper is a major modulator of germination, development and secondary metabolism in *Streptomyces coelicolor*

**DOI:** 10.1038/s41598-019-40876-0

**Published:** 2019-03-12

**Authors:** Nathaly González-Quiñónez, Mario Corte-Rodríguez, Roberto Álvarez-Fernández-García, Beatriz Rioseras, María Teresa López-García, Gemma Fernández-García, María Montes-Bayón, Angel Manteca, Paula Yagüe

**Affiliations:** 1 Área de Microbiología, Departamento de Biología Funcional, IUOPA and ISPA, Facultad de Medicina, Universidad de Oviedo, 33006 Oviedo, Spain; 2 Department of Physical and Analytical Chemistry, Faculty of Chemistry and ISPA, Universidad de Oviedo, 33006 Oviedo, Spain

## Abstract

Streptomycetes are important biotechnological bacteria with complex differentiation. Copper is a well-known positive regulator of differentiation and antibiotic production. However, the specific mechanisms buffering cytosolic copper and the biochemical pathways modulated by copper remain poorly understood. Here, we developed a new methodology to quantify cytosolic copper in single spores which allowed us to propose that cytosolic copper modulates asynchrony of germination. We also characterised the SCO2730/2731 copper chaperone/P-type ATPase export system. A *Streptomyces coelicolor* strain mutated in *SCO2730/2731* shows an important delay in germination, growth and sporulation. Secondary metabolism is heavily enhanced in the mutant which is activating the production of some specific secondary metabolites during its whole developmental cycle, including germination, the exponential growth phase and the stationary stage. Forty per cent of the *S. coelicolor* secondary metabolite pathways, are activated in the mutant, including several predicted pathways never observed in the lab (cryptic pathways). Cytosolic copper is precisely regulated and has a pleiotropic effect in gene expression. The only way that we know to achieve the optimal concentration for secondary metabolism activation, is the mutagenesis of *SCO2730/2731*. The *SCO2730/2731* genes are highly conserved. Their inactivation in industrial streptomycetes may contribute to enhance bioactive compound discovery and production.

## Introduction

Streptomycetes are important biotechnological bacteria from which two thirds of the bioactive secondary metabolites used in clinic (mainly antibiotics, but also antitumorals, immunosupressors, etc.) were discovered^1,2^. They have a complex developmental cycle that makes this bacterium a multicellular prokaryotic model including programmed cell death (PCD) and hyphae differentiation, which leads to aerial mycelium formation and sporulation^3,4^.

One of the less studied stages of *Streptomyces* development is spore germination. There are proteomic and transcriptomic works demonstrating that spore germination is highly regulated (reviewed in Bobek *et al*.^5^). However, the biomolecular mechanisms controlling germination remain poorly characterised^6,7^. There are some proteins known to be involved in *Streptomyces* spore germination: NepA, a structural cell wall protein involved in the maintenance of spore dormancy in *S. coelicolor*^8^; SsgA, a protein marking cell-wall sites where germination takes place^9^; resuscitation-promoting factors (Rpfs), cell wall hydrolases^10^ controlling germination; OsdR, a *Streptomyces* orthologue to the *M. tuberculosis* DevR dormancy regulator, which was demonstrated to be functional in *Mycobacterium*^11^; and SCO4439, a D-alanyl-D-alanine carboxypeptidase that controls spore peptidoglycan crosslinking and conditions spore germination^12^. One of the most intriguing and poorly known aspects of spore germination is asynchrony i.e. some spores germinate early, while others take a long time to germinate or even do not germinate^5^. To the best of our knowledge, NepA is the only known protein that contributes to this asynchrony^8^.

Copper has been characterised as a positive regulator of *Streptomyces* differentiation (aerial mycelium, sporulation) and antibiotic production^13,14^. By contrast, vegetative growth was described to be unaffected by copper^14^. At high concentrations (over 750 µM), the positive effect in aerial mycelium development and sporulation becomes a negative effect (delay)^15^. The mechanisms controlling copper trafficking in *Streptomyces* remains poorly understood. Worrall and Vijgenboom^16^ predicted the existence of two copper chaperone/P-type ATPases (CopZ/CopA) modulating copper export (SCO1045/1046, SCO2730/2731). They demonstrated that the expression of the genes encoding these transporters is regulated by CsoR, the master transcriptional repressor modulating copper effects in gene transcription^16^. This research group also demonstrated that the number of genes responding to copper stress is much higher than those regulated by CsoR. Dwarakanath *et al*.^15^ proposed a model in *S. lividans* describing the copper effect modulating CsoR activity and expression of the *SCO2730/2731* and *SCO1045/1046* copper chaperones/transporters: under “normal” cytosolic copper concentration, *csoR* and *SCO2730* (*copZ*) are expressed at a low level; when cytosolic copper concentration increases, CsoR and SCO2730 (CopZ) buffer copper at first, but as soon they become saturated, the CsoR repression of *SCO2730/2731* and *SCO1045/1046* is unblocked, increasing the expression of these copper secretion transporters that maintain copper homeostasis. Interestingly, CopZ-3079 (the *S. lividans SCO2730* orthologue) was demonstrated *in vitro* to have a 5-fold higher affinity for Cu than CopZ-1317 (the SCO1045 orthologue)^17^, indicating that its physiological relevance in copper trafficking is higher at low copper concentrations, as proposed in the Dwarakanath *et al*. model^15^.

The copper import mechanisms in *Streptomyces* remain basically unknown. The existence of a membrane-bound periplasmic cupric reductase and a P-type ATPase importing Cu(I) has been postulated^18^.

In this work we focus on the characterisation of the SCO2730/SCO2731 copper export system. *SCO2730* encodes for a putative copper chaperone^15^, highly conserved in the *Streptomyces* genus. *SCO2731* encodes a conserved putative P-type ATPase, which was predicted to transport the copper carried out by the *SCO2730* chaperone^15^. Our results contribute to the knowledge of complex pleiotropic effects of copper in *Streptomyces* development, including important effects regulating the asynchrony of germination, vegetative growth and activation of secondary metabolism, which were previously un-described.

## Results

### *SCO2730/*2731 are highly conserved in *Streptomyces*

**Figure 1:**
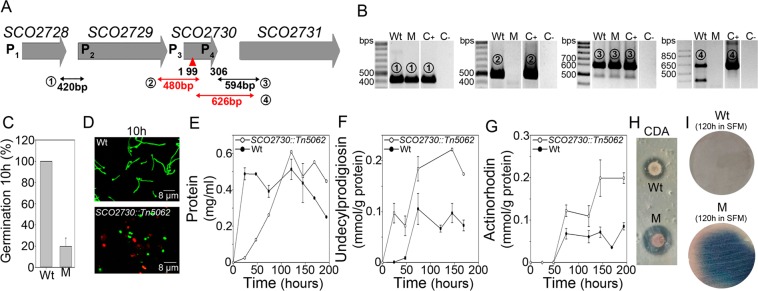
*SCO2730/2731* expression and phenotype of the *SCO2730::Tn5062* mutant. (**A**) Outline of the *SCO2728-2731* chromosomal region illustrating the position of the primers used for RT-PCR. Fragments not amplified in the *SCO2730::Tn5062* mutant are outlined in red. (**B**) RT-PCRs demonstrating co-transcription of the following: SCO2728 and SCO2729 (1), SCO2729 and SCO2730 (2), SCO2730 and SCO2731 (3 and 4). Full-length gels are shown in SI Fig. S2. (**C**) Percentage of germination in the mutant (M) and the *S. coelicolor* wild-type strain (Wt) at 10 h. (**D**) Confocal microscope images of the mutant and the wild-type strain stained with SYTO9 (green) and propidium iodide (red) at 10 h. (**E**) Growth curves (three biological replicates). (**F**) Undecylprodigiosin production (three biological replicates). (**G**) Actinorhodin production (three biological replicates). (**H**) CDA production. (**I**) Sporulation (grey colour) in SFM medium.

SCO2730 and SCO2731 show an average amino acid similarity of 88.7% and 81% respectively, among *S. griseus*, *S. avermitillis*, *S. lividans*, *S. clavuligerus* and *S. venezuelae*. A comparison of *SCO2730* orthologues reveal that the *SCO2730* ORF probably starts at position 66 (ATG, Met) instead of the GTG (Val), annotated in the StrepDb database (http://strepdb.streptomyces.org.uk/). The *S. clavuligerus* *SCO2731* orthologue is not annotated in the StrepDb database, but is present downstream of *SCLAV_1906*, the *SCO2730* orthologue (data not shown). The *SCO2730/2731* orthologues are together in all of the *Streptomyces* chromosomes analysed. However, the *SCO2728*–*SCO2731* synteny (Fig. 1A) is only present in *S. coelicolor* and *S. lividans*.

### The *SCO2730* mutation affects spore germination, antibiotic production and sporulation

Cosmid C46.2.D06 was used to obtain the *S. coelicolor SCO2730::Tn5062* mutant harbouring *SCO2730* interrupted by *Tn506*2^19^. RT-PCR demonstrated that *SCO2730* and *SCO2731* are co-transcribed in the *S. coelicolor* wild-type strain (amplicons 3 and 4 in Fig. 1A,B). The *SCO2731* expression was affected in the *SCO2730::Tn5062* mutant (amplicon 4 in Fig. 1B). Surprisingly, amplicon 3 was present in the mutant (Fig. 1B), indicating the existence of a promoter (promoter 4 in Fig. 1A) between the two forward oligonucleotides used for RT-PCR 3 and 4. RT-PCR also revealed that *SCO2730* was co-transcribed with *SCO2729* (amplicon 2 in Fig. 1B) and that *SCO2728* was co-transcribed with *SCO2729*, suggesting that all of these genes might be transcribed as a single operon.

Spore germination was dramatically delayed in the *SCO2730::Tn5062* mutant, compared to the wild-type strain (19.5% in the mutant vs. 100% in the wild-type strain at 10 h in sucrose-free R5A medium) (Fig. 1C). In solid sucrose-free R5A, the *S. coelicolor* wild-type strain reaches 100% of the germination at approximately 8 hours. However, as germination is highly delayed in the *SCO2730::Tn5062* mutant, in this study we show germination at 10 hours. Moreover, there was a high rate of cell death during germination in the *SCO2730::Tn5062* mutant (propidium iodide staining, red colour in Fig. 1D, 10 h). Growth was highly delayed in the mutant (Fig. 1E), while undecylprodigiosin and actinorhodin production was doubled (Fig. 1F,G). Calcium dependent antibiotic (CDA) production was slightly increased in the *SCO2730::Tn5062* mutant (Fig. 1H). The *SCO2730::Tn5062* mutant produced undecylprodigiosin starting with the first time point analysed (24 hours) and indicated that secondary metabolism is accelerated in this mutant (Fig. 1F). Sporulation was highly delayed and reduced in the *SCO2730::Tn5062* mutant (Fig. 1I).

### The *SCO2730::Tn5062* mutant phenotype in germination and antibiotic production depend on the alteration of *SCO2730* and *SCO2731* expression

**Figure 2:**
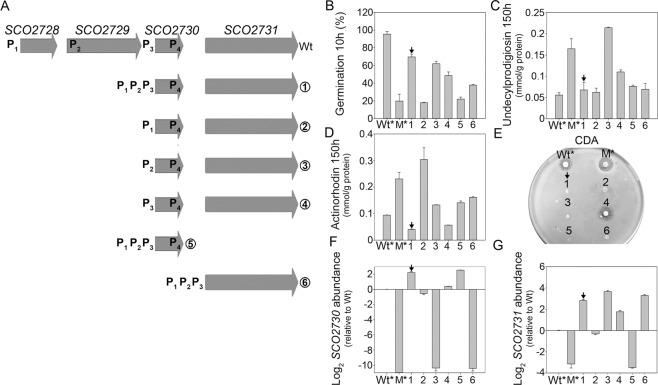
Complementation of the wild-type phenotype in the *SCO2730::Tn5062* mutant. (**A**) Outline of the *SCO2728-2731* chromosomal region and the six complementation constructions created. Promoter localisations are indicated. (**B**) Percentage of germination at 10 hours. (**C**) Undecylprodigiosin production at 150 hours. (**D**) Actinorhodin production at 150 hours. (**E**) CDA production. (**F**) *SCO2730* expression (qRT-PCR, two biological replicates). (**G**) *SCO2731* expression (qRT-PCR, two biological replicates). Wt: *S. coelicolor* wild-type strain. Wt^*^: *S. coelicolor* harbouring pNG3. Arrows indicate complementation of the phenotype in the *SCO2730::Tn5062* mutant harbouring *SCO2730/2731* under the control of promoters P_1_,P_2_ and P_3_. M^*^: *SCO2730::Tn5062* mutant harbouring pNG3.

Based on the above results, we proceeded to identify the gene/s responsible for the phenotypes detected. We complemented the *SCO2730::Tn5062* mutant with different combinations of the three promoters located upstream of *SCO2730*, combined with the *SCO2730* and/or *SCO2731* ORFs (strains 1–6 in Fig. 2A). The *SCO2730::Tn5062* mutant strains harbouring the different promoters, together with *SCO2730* and/or *SCO2731*, complemented part of the germination (measured at 10 hours) (Fig. 2B) or antibiotic production phenotypes (Fig. 2C–E). Undecylprodigiosin and actinorhodin production was measured at 150 hours, when the maximum production was reached, while CDA was measured in solid plates (see Methods). Only the *SCO2730::Tn5062* mutant strain harbouring the three promoters and the *SCO2730/2731* ORFs (strain 1) complemented the *SCO2730::Tn5062* mutant phenotype in germination (Fig. 2B) and antibiotic production (Fig. 2C–E). Undecylprodigiosin production was restored to the wild-type level. However, the wild-type phenotype was not fully restored. Germination was accelerated in complemented strain 1 compared to the *SCO2730::Tn5062* mutant (notice that germination values in Fig. 2B are out of the average ± SD confidence intervals), but strain 1 germination did not reach the wild-type level (Fig. 2B); actinorhodin and CDA production was reduced in strain 1 compared to the mutant (actinorhodin production was out of the average ± SD confidence intervals in Fig. 2D; CDA production was not detectable in strain 1 in Fig. 2E), but the production of both antibiotics was less than that of the wild-type strain.

The expression of the *SCO2730* and *SCO2731* genes was not restored in any of the complemented strains (Fig. 2F,G), indicating the existence of further unknown regulation beyond the three promoters considered in this work and explaining why the wild type phenotype was not restored in the complementation strains. The *SCO2730/2731* genes were overexpressed in the mutant strain harbouring the three promoters and the *SCO2730/2731* ORFs (strain 1 in Fig. 2F,G). Interestingly, all of the *SCO2730::Tn5062* complemented strains that overexpressed *SCO2731* (strains 1, 3, 4 and 6) (Fig. 2G) showed an increase in germination (Fig. 2B) compared to the *SCO2730::Tn5062* mutant.

### *SCO2730* and *SCO2731* expression correlates with copper secretion during germination

**Figure 3:**
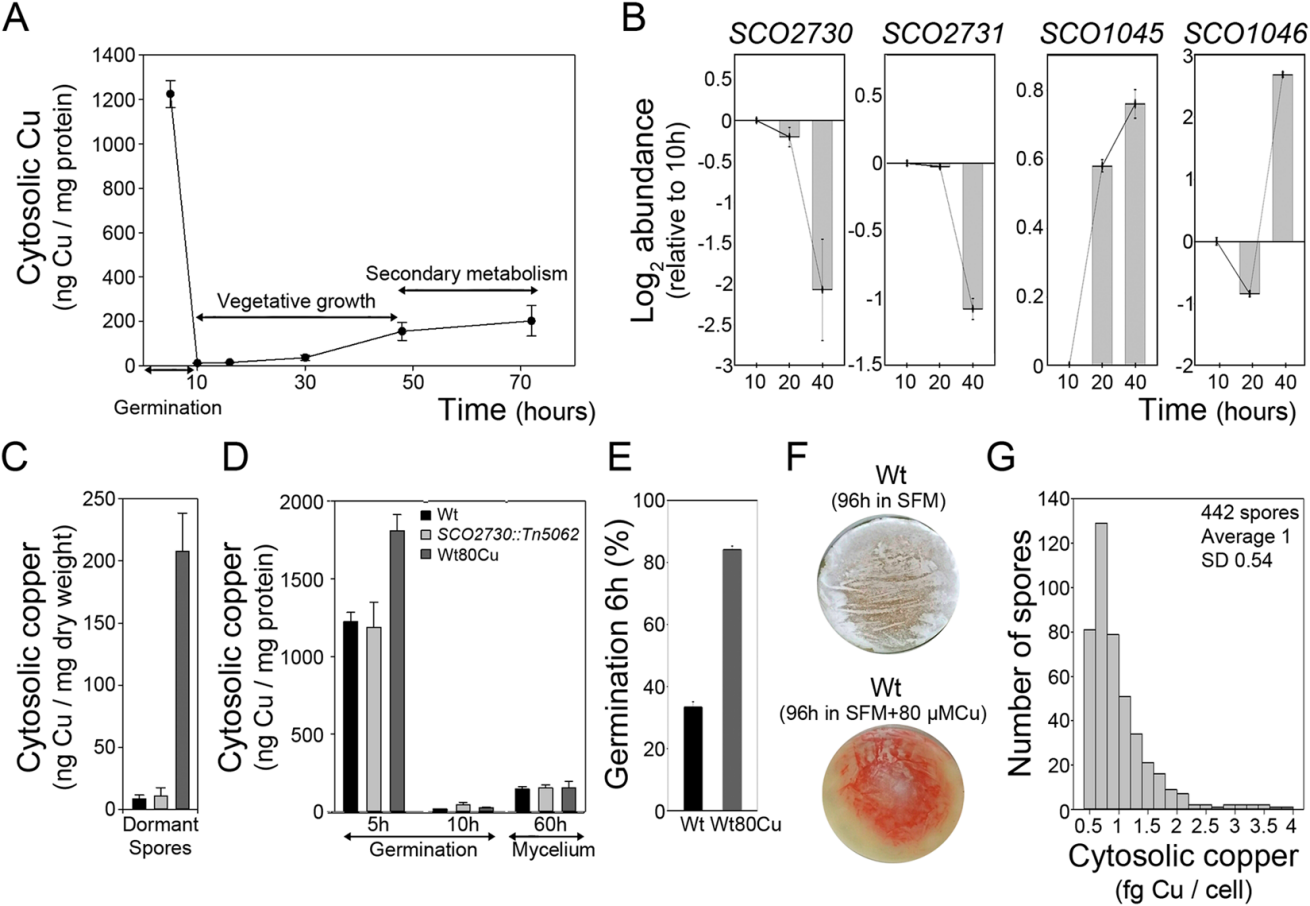
Cytosolic copper and *SCO2730/2731* expression during *S. coelicolor* development. (**A**) Cytosolic copper during development in sucrose free R5A liquid cultures. Vegetative growth and secondary metabolism time windows are indicated. (**B**) *SCO2730/2731* and *SCO1045/1046* expression during development (qRT-PCRs, two biological replicates). (**C**) Cytosolic copper in dormant spores (three biological replicates). (**D**) Cytosolic copper in germinating spores (5- and 10 h) and mycelium (60 h) (three biological replicates). (**E**) Percentage of germination (three biological replicates) in Wt and Wt80Cu. (**F**) Macroscopic view of sporulation (grey colour) of the wild-type strain in SFM and SFM amended with 80 µM CuSO_4_. (**G**) Histogram showing the cytosolic copper in single spores of the wild type strains obtained in 80 µM CuSO_4_ copper amended SFM cultures. Wt, wild-type strain. Wt80Cu, spores of the wild-type strain obtained in 80 µM CuSO_4_ amended SFM cultures.

We analysed the cytosolic copper concentration and the expression of the genes encoding the two copper secretion systems described in *S. coelicolor* (*SCO2730/2731* and *SCO1045/1046*) during development^15^ (germinated spores, 5–10 h; mycelium, 20–70 h) (Fig. 3A,B). Cytosolic copper (normalised against cytosolic protein) decreases quickly during germination (Fig. 3A). The maximum levels of *SCO2730/2731* expression were reached at 10 hours, coinciding with the lowest cytosolic copper concentration and the lowest expression of *SCO1045/1046* (Fig. 3B). This result suggests that SCO2730/2731 chaperone/transport system is the main effector in copper secretion during germination.

Next, we analysed cytosolic copper in dormant spores, germinated spores (5 and 10 hours) (Fig. 3C) and mycelium (60 h) of the *SCO2730::Tn5062* mutant and the *S. coelicolor* wild-type strains (Fig. 3D). Dormant spores accumulate high amounts of trehalose and glycogen^20^ that are not present in germinated spores or mycelia. This makes normalisation and comparison of cytosolic copper concentrations between dormant and germinated spores/mycelia, a challenge. Cytosolic copper was normalised in dormant spores against dry weight, while in germinated spores and mycelium, it was normalised against cytosolic protein (see Methods). Consequently, copper abundances can be compared between dormant spores (Fig. 3C) or between germinated spores and the mycelium (Fig. 3D), but not between dormant spores and germinated spores/mycelium. In the case of the wild-type strain we analysed spores obtained in SFM solid cultures (Wt) and spores obtained in SFM solid cultures amended with 80 µM Cu (Wt80Cu). Cytosolic copper concentration was similar in the *SCO2730::Tn5062* mutant and in the Wt dormant spores (11 ± 6 and 8.5 ± 3 ng Cu/mg dry weight respectively) but it was much higher in the Wt80Cu spores (208 ± 30, 24-fold higher than in the wild-type strain) (Fig. 3C). Cytosolic copper dropped dramatically during germination in copper unmodified sucrose-free R5A cultures of the wild-type strain inoculated with Wt or Wt80Cu spores (66-fold and 70- fold drop respectively from 5 to 10 hours) (Fig. 3D), but the reduction in cytosolic copper was lesser in the cultures inoculated with *SCO2730::Tn5062* mutant spores (26-fold drop from 5 to 10 hours) (Fig. 3D). Cytosolic copper was higher at 10 hours in the *SCO2730::Tn5062* mutant (45.7 ± 5.4 ng Cu/mg protein) compared to the wild-type strain (cultures using Wt or Wt80Cu spores) (18.6 ± 1.6 and 24.2 ± 2.4 ng Cu/mg protein respectively) (Fig. 3D). By contrast, cytosolic copper in the mycelium of the *SCO2730::Tn5062* mutant (153 ± 20 ng Cu/mg protein) and the wild-type strains (145.7 ± 18 and 155.4 ± 41 ng Cu/mg protein in Wt and Wt80Cu spores respectively) are comparable (Fig. 3D).

Interestingly, germination was highly accelerated in the Wt80Cu spores compared to the Wt spores (both growing in copper unmodified cultures) (Fig. 3E, 6 hours culture), while growth and antibiotic production were similar (data not shown). Germination starts at comparable time points in the Wt and the Wt80Cu spores (data not shown), but it is much more synchronous in the Wt80Cu than in the Wt, which lead to an acceleration in the time point at which 100% germination is reached (Fig. 3E). Sporulation in the SFM cultures adjusted with 80 µM Cu was delayed compared to the SFM unmodified cultures (Fig. 3F), confirming the effect of copper modulation of sporulation reported before^15^.

### Cytosolic copper quantification in single spores

To analyse cytosolic copper in individual spores, we adapted the method that we developed for the analysis of single eukaryotic cells^21^ to the study of *Streptomyces* single spores. Cytosolic copper in the Wt and in the *SCO2730::Tn5062* mutant single spores was below the sensitivity of the analysis (see Methods). However, we were able to quantify cytosolic copper in the Wt80Cu spores (Fig. 3G). There was a high variability in the cytosolic copper with a minimum of 0.51 fg Cu per spore and a maximum of 3.83 fg Cu per spore.

### Cytosolic copper modulates spore germination and *SCO2730/2731–SCO1045/1046* gene expression

**Figure 4:**
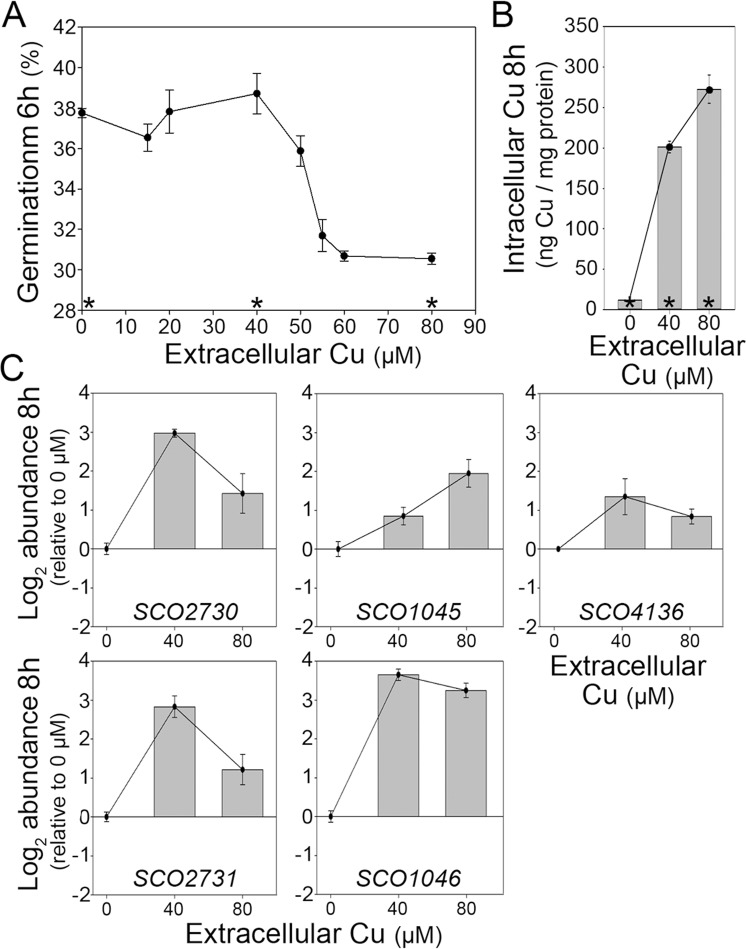
Extracellular copper effect in germination, cytosolic copper levels and the expression of copper related genes. (**A**) Variation of the percentage of germination with the extracellular copper concentration in sucrose free R5A solid cultures (three biological replicates). (**B**) Variation of intracellular copper during germination (6 h) depending on the extracellular copper concentration in sucrose free R5A liquid cultures (three biological replicates). (**C**) *SCO2730/2731*, *SCO1045/1046* and *SCO4136* expression during germination (8 h) in 40 and 80 µM CuSO_4_ amended cultures (qRT-PCRs, two biological replicates, sucrose free R5A liquid medium).

The above results suggest an effect of *SCO2730* and *SCO2731* in the modulation of cytosolic copper concentration and germination (Figs 1 and 3). We next analysed the effect of copper on germination. Germination was delayed in sucrose-free R5A liquid cultures treated with copper concentrations over 40 µM (Fig. 4A). Interestingly, cytosolic copper was not proportional to the copper added to the medium. An increase of 2-fold in the extracellular copper (from 40 to 80 µM) correlated with a 1.3-fold increase of cytosolic copper (from 201.3 ± 7.4 to 272.5 ± 38.8 ng/mg protein) (Fig. 4B).

In order to further study the biomolecular mechanism buffering cytosolic copper during germination, we analysed the expression of the two copper secretion systems reported in *Streptomyces* (*SCO2730/2731*, *SCO1045/1046*)^15^ during germination in copper modified (by adding 0-, 40- or 80 µM extracellular copper) sucrose-free R5A cultures. The *SCO2730/2731* maximum expression (compared to the Wt germinating spores at the 0 µM copper modified medium) was reached at 40 µM extracellular copper, while the maximum expression of the *SCO1045/1046* genes was reached at 80 µM copper (Fig. 4C). We further analysed the expression of *csoR* (*SCO4136*), the master transcriptional repressor modulating copper effects in gene transcription^15^. *CsoR* expression was equally activated during germination at high cytosolic copper concentration, which was reached in the 40 and 80 µM copper modified sucrose-free R5A cultures (Fig. 4C). As reported before^15^, copper binds to CsoR blocking its interaction with DNA and activating the expression of genes repressed by CsoR, including the *csoR* gene.

### Comparison of the transcriptomes of *SCO2730::Tn5062* and *S. coelicolor* wild-type Cu-amended/non-amended spores, during germination

**Figure 5:**
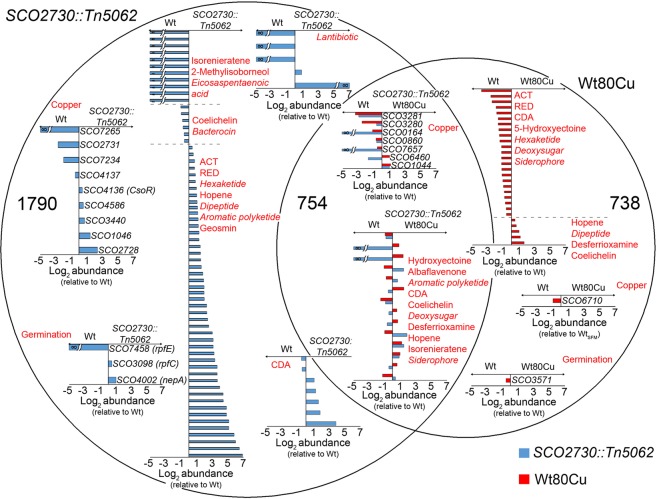
Transcriptomic analysis of the *SCO2730::Tn5062* mutant spores, the *S. coelicolor* wild-type strain spores obtained in 80 µM CuSO_4_ amended SFM cultures (Wt80Cu) and in SFM cultures (Wt) during germination (10 h) in sucrose free R5A medium. The Venn diagram shows transcripts related to copper homeostasis/metabolism/regulation, germination and secondary metabolism with significant variations (q-value of less than 0.05) in the *SCO2730::Tn5062* mutant (1790 transcripts), the Wt80Cu strain (738 transcripts), or both (754 transcripts), compared to the Wt. Abundance values (average from two biological replicates) are shown as log2 (*SCO2730::Tn5062* or Wt80Cu/Wt). Blue bars: transcripts with significant variations in the *SCO2730::Tn5062* mutant compared to Wt. Red bars, transcripts with significant variations in the Wt80Cu compared to Wt. Broken bars indicate transcripts not detected in the *SCO2730::Tn5062* mutant or the Wt strain. ACT, actinorhodin; RED undecylprodigiosin; CDA, calcium dependent antibiotic. Secondary metabolism cryptic pathways are indicated in italic letters.

In order to investigate the molecular mechanisms controlling the phenotypes observed during germination of the *SCO2730::Tn5062* mutant and the Wt80Cu spores, we compared their transcriptomes during germination (10 hours) in sucrose-free R5A liquid medium. Data were normalised against the Wt spores (log2 *SCO2730::Tn5062* or Wt80Cu/Wt) (SI Table S1). 1790 transcripts showed significant variations (q-value of less than 0.05) in the *SCO2730::Tn5062* mutant, 738 in the Wt80Cu and 754 in both, *SCO2730::Tn5062* and Wt80Cu (Fig. 5) (SI Table S1).

The 1790 genes differentially expressed in the *SCO2730::Tn5062* mutant compared to the Wt strain, included key secondary metabolism genes, spore germination genes and genes encoding for copper related proteins (copper transporters, proteins using copper as cofactor, proteins whose genes are known to be regulated by copper) (Fig. 5) (Table 1). Most of the genes involved in secondary metabolism were up-regulated in the *SCO2730::Tn5062* mutant during germination (see details in the next paragraph). The expression of *SCO1046* (CopA)^15^, *SCO4136* (CsoR)^15^, *SCO4586* (ABC transporter involved in multi-copper enzyme maturation, conserved domain database accession COG1277) and *SCO3440* (oxidase predicted to bind to copper)^16^ were up-regulated in the mutant. The expression of *SCO4137* and *SCO7265* (two genes predicted to be under the control of CsoR)^15^, *SCO7234* (cytochrome c oxidase predicted to use copper as cofactor, PROSITE accession PS00077) and *SCO2731* (cation transporter)^15^, was down-regulated in the mutant. As mentioned above, *SCO2731* was expressed in the *SCO2730::Tn5062* mutant (amplicon 3 in Fig. 1), from a promoter located inside the *SCO2730* ORF, but downstream of the *Tn5062* insertion. However, the expression from this promoter is 6.5-fold less than the wild-type strain (Table 1). Interestingly, genes *SCO2728* (putative zinc-responsive transcriptional regulator, conserved domain database accession PRK09514) and *SCO2729* (putative acetyltransferase), both transcribed together with *SCO2730/3731* (Fig. 1A), are 5-fold overexpressed in the mutant (Table 1). Three genes related to spore germination are differentially expressed in the mutant and the wild-type strain: *nepA* (*SCO4002*)^8^, 2-fold up-regulated; *rpfC* (*SCO3098*)^10^, 1.4-fold up-regulated; and *rpfE* (*SCO7458*), encoding one of the key peptidoglycan lytic enzymes activated during germination^10^, whose transcript was absent in the mutant, and perhaps contributing to the delay in germination observed in the mutant (Table 1).

**Table 1:** Abundance values of transcripts showing significant variations (q-value of less than 0.05) between the *SCO2730::Tn5062* mutant and the *S. coelicolor* M145 wild-type strain during germination (10 hours) in sucrose free R5A cultures.

Function	Sco No.	Description	Abundance	
			Log_2_ (*SCO2730::Tn5062* /Wt)	*SCO2730::Tn5062*/Wt
Copper related genes	*SCO1046*	Copper transporter	1.4	2.7
	*SCO2731*	CopA cation transporter	−2.7	0.1
	*SCO3440*	Oxidase predicted to bind to copper	0.7	1.6
	*SCO4137*	Putatively repressed by CsoR	−0.5	0.7
	*SCO4136*	CsoR	0.4	1.3
	*SCO4586*	ABC transporter involved in multi-copper enzyme maturation	0.5	1.4
	*SCO7234*	Cytochrome c oxidase	−2	0.2
	*SCO7265*	Putatively repressed by CsoR	^a^	0
Genes upstream SCO2730	*SCO2728*	Putative zinc-responsive transcriptional regulator	2.4	5.1
	*SCO2729*	Putative acetyltransferase	2.2	4.7
Germination	*SCO4002*	*NepA*	1	2
	*SCO3098*	*RpfC*	0.4	1.4
	*SCO7458*	*RpfE*	^a^	0
Secondary metabolism	*SCO0124*	Eicosapentaenoic acid biosynthesis	^a^	0
	*SCO0126*		^a^	0
	*SCO0127*		^a^	0
	*SCO0129*		^a^	0
	*SCO0186*	Isorenieratene biosynthesis	^a^	0
	*SCO0187*		^a^	0
	*SCO0188*		^a^	0
	*SCO0189*		^a^	0
	*SCO0190*		^a^	0
	*SCO0267*	Lantibiotic biosynthesis	^a^	0
	*SCO0269*		^a^	0
	*SCO0270*		^a^	0
	*SCO0490*	Coelichelin biosynthesis	−0.6	0.7
	*SCO0491*		−0.6	0.7
	*SCO0496*		−1	0.5
	*SCO0497*		−1	0.5
	*SCO0499*		−0.8	0.6
	*SCO0756*	Bacteriocin biosynthesis	−0.8	0.6
	*SCO1271*	Aromatic polyketide biosynthesis	0.8	1.7
	*SCO1909*	Antibiotic biosynthesis monooxygenase	1.1	2.2
	*SCO3216*	CDA biosynthesis	−0.6	0.6
	*SCO3217*		1	2
	*SCO3220*		−0.5	0.7
	*SCO3222*		1.8	3.5
	*SCO3236*		3.8	14.4
	*SCO3247*		1.2	2.4
	*SCO3248*		1.6	3
	*SCO3800*	Acyl-CoA dehydrogenase	2	3.8
	*SCO5071*	ACT biosynthesis	3.7	12.6
	*SCO5072*		3	8
	*SCO5073*		1.9	3.9
	*SCO5074*		2.6	5.9
	*SCO5075*		1.3	2.5
	*SCO5076*		0.4	1.4
	*SCO5078*		1.5	2.8
	*SCO5080*		2.3	4.9
	*SCO5082*		1.5	2.8
	*SCO5083*	ACT biosynthesis	4.4	21.5
	*SCO5084*		3.9	15.7
	*SCO5085*		3.6	12.5
	*SCO5086*		3.2	9.4
	*SCO5087*		2	4
	*SCO5088*		1.8	3.5
	*SCO5090*		2.3	4.9
	*SCO5091*		1.1	2.2
	*SCO5092*		1.2	2.3
	*SCO5877*	Prodigiosin biosynthesis	0.9	1.8
	*SCO5879*		1.3	2.5
	*SCO5890*		0.6	1.5
	*SCO5895*		1.5	2.9
	*SCO6073*	Geosmin biosynthesis	1.9	3.7
	*SCO6273*	Hexaketide biosynthesis	3.7	12.5
	*SCO6274*		3.1	8.7
	*SCO6275*		4.8	28.6
	*SCO6276*		6	64.1
	*SCO6277*		1.6	3
	*SCO6278*		5.1	35.3
	*SCO6279*		5.8	55.8
	*SCO6280*		2.4	5.4
	*SCO6281*		4.8	28.7
	*SCO6282*		6.9	118.9
	*SCO6283*		6.5	90.4
	*SCO6284*		5.1	34.9
	*SCO6286*		3.4	10.2
	*SCO6287*		4.4	21.1
	*SCO6288*		3.1	8.7
	*SCO6430*	Dipeptide biosynthesis	1.2	2.3
	*SCO6431*		1	2
	*SCO6432*		0.9	1.8
	*SCO6436*		0.8	1.7
	*SCO6767*	Hopene biosynthesis	1	2
	*SCO6927*	Lantibiotic biosynthesis	0.8	1.8
	*SCO6932*		^b^	∞
	*SCO7700*	2-Methylisoborneol biosynthesis	^b^	∞
	*SCO7701*		^b^	∞

The abundance of the transcripts described in results is indicated; see SI Table S1 for the full transcriptome results. ^a^Transcripts not detected in the *SCO2730::Tn5062* mutant.

^b^Transcripts not detected in the Wt strain.

The 738 genes differentially expressed during the germination of the Wt80Cu spores compared to Wt, include key genes involved in secondary metabolism, germination and genes encoding for copper related proteins (Fig. 5) (Table 2). The expression of most of the genes involved in secondary metabolite biosynthesis was down-regulated in the cultures inoculated with Wt80Cu spores (genes involved in actinorhodin, undecylprodigiosin, CDA, 5-hydroxyectoine, hexaketide, deoxysugar and siderophore biosynthesis). Other genes down-regulated in the Wt80Cu cultures were: *SCO6710*, a putative glycosyl hydrolase supposedly repressed by CsoR^15^; and *SCO3571*, a cyclic AMP receptor protein homologue whose mutation was reported to diminish germination^22^. There were no significant differences in *csoR* between Wt80Cu and the Wt, indicating that inactivation of the CsoR repressor activity triggered by copper binding previously described^15^ did not occur at the high cytosolic copper levels present in the Wt80Cu dormant spores (Fig. 3C). Consequently, there remains an unknown CsoR independent mechanism controlling the fast spore germination observed in this strain (Fig. 3E). Interestingly, neither of the two copper chaperones/transporters described in *Streptomyces* (SCO1045/1046 and SCO2730/2731)^15^ showed significant variations in the Wt80Cu cultures compared to the Wt cultures (Table 2), indicating that the Wt expression levels present in the Wt80Cu spores are high enough to reduce cytosolic copper during germination to a concentration comparable to that reached in the Wt spores (26 ± 2.4 ng Cu/mg protein, only 1.4- fold higher than the Wt spores at 10-hours) (Fig. 3D). The expression of genes involved in hopene, desferrioxamine, coelichelin and dipeptide biosynthesis, was slightly up-regulated in the Wt80Cu cultures.

**Table 2:** Abundance values of transcripts in the Wt80Cu and the *S. coelicolor* M145 wild-type strains during germination (10 hours) in sucrose free R5A cultures.

Function	Sco No.	Description	Abundance	
			Log _2_ (Wt80Cu /Wt)	Wt80Cu/Wt
Copper related genes	*SCO2730*	CopZ copper chaperone	n.s.	n.s.
	*SCO2731*	CopA cation transporter	n.s.	n.s.
	*SCO1045*	CopZ copper chaperone	n.s.	n.s.
	*SCO1046*	CopA cation transporter	n.s.	n.s.
	*SCO6710*	Glycosyl hydrolase putatively repressed by CsoR	−1	0.5
	*SCO4136*	CsoR	n.s.	n.s.
Germination	*SCO3571*	Cyclic AMP receptor protein homologue	−0.6	0.7
Secondary metabolism	*SCO0381*	Deoxysugar synthase	−1.5	0.3
	*SCO0382*		−1.3	0.1
	*SCO0384*		−1.9	0.2
	*SCO0385*		−1.3	0.4
	*SCO0386*		−0.9	0.5
	*SCO0387*		−1.7	0.3
	*SCO0388*		−1	0.5
	*SCO0389*		−1.1	0.5
	*SCO0395*		−2.5	0.2
	*SCO0397*		−1.6	0.3
	*SCO0399*		−1.5	0.4
	*SCO0493*	Coelichelin biosynthesis	0.5	1.4
	*SCO1864*	5-Hydroxyectoine biosynthesis	−0.8	0.6
	*SCO1865*		−1.2	0.4
	*SCO1866*		−1.2	0.4
	*SCO2783*	Desferrioxamine biosynthesis	1	2
	*SCO2785*		0.6	1.6
	*SCO3235*	CDA biosynthesis	−0.6	0.6
	*SCO3774*	Beta-lactamase related protein	−0.8	0.6
	*SCO5800*	Siderophore biosynthesis	−1.3	0.4
	*SCO5881*	Prodigiosin biosynthesis	−0.7	0.6
	*SCO6275*	Hexaketide biosynthesis	−0.9	0.5
	*SCO6277*		−1.3	0.4
	*SCO6279*		−1.7	0.3
	*SCO6280*		−2	0.2
	*SCO6282*		−3.8	0.07
	*SCO6283*		−2.6	0.2
	*SCO6437*	Dipeptide biosynthesis	1.1	2.2
	*SCO6770*	Hopene biosynthesis	1.6	3.1

The abundance of the transcripts described in results is indicated; see SI Table S1 for the full transcriptome results. N.s. Non-significant variation.

The 754 genes differentially expressed during germination in both the *SCO2730::Tn5062* mutant and the cultures inoculated with the Wt80Cu spores, compared with the wild-type strain, include secondary metabolism genes, and genes encoding for copper related proteins (Fig. 5) (Table 3). Most of these genes were down-regulated in the cultures inoculated with Wt80Cu spores. Variation in the expression of secondary metabolism genes was discrete (all the abundances were below the 2-fold change, log2 abundance within the +/− 1 interval). The most important variation, was found in the genes encoding proteins related to copper: genes predicted to be modulated by CsoR (*SCO1044*, *SCO3280*)^15^; putative cation-transporting ATPases (*SCO0164*, *SCO0860*, *SCO6460*)^15^; *SCO3281*, a cytosolic copper storage protein^23^; and *SCO7657*, a putative secreted protein predicted to bind copper^16^. Interestingly, *SCO0164* and *SCO7657* were not expressed in the *SCO2730::Tn5062* mutant.

**Table 3:** Abundance values of transcripts showing significant variation (q-value of less than 0.05) in the *SCO2730::Tn5062* mutant and the Wt80Cu strain compared to the *S. coelicolor* M145 wild-type strain during germination (10 hours) in sucrose free R5A cultures.

Function	Sco No.	Description	Abundance			
			Log _2_ (*SCO2730::Tn5062* /Wt)	Log _2_ (Wt80Cu /Wt)	*SCO2730::Tn5062* /Wt	Wt80Cu /Wt
Copper related genes	*SCO0164*	Putative cation-transporting ATPase	^a^	−1.2	0	0.4
	*SCO0860*	Putative cation-transporting ATPase	−0.7	−0.7	0.6	0.6
	*SCO1044*	Putatively repressed by CsoR	1	1.1	2	2.2
	*SCO3280*	Putatively repressed by CsoR	−0.7	−2.5	0.6	0.2
	*SCO3281*	Cytosolic copper storage protein	−2.9	−3.3	0.1	0.09
	*SCO6460*	Putative cation-transporting ATPase	−1.7	1	0.3	2
	*SCO7657*	Putative secreted protein predicted to bind copper	^a^	−0.6	0	0.7
Secondary metabolism	*SCO0185*	Isorenieratene biosynthesis	^a^	1.4	0	2.6
	*SCO0191*		^a^	0.8	0	1.8
	*SCO0396*	Deoxysugar synthase	−0.9	−1.5	0.5	0.3
	*SCO0494*	Coelichelin biosynthesis	−0.5	0.5	0.7	1.4
	*SCO0495*	Coelichelin biosynthesis	−0.7	0.6	0.6	1.6
	*SCO1265*	Aromatic polyketide biosynthesis	0.8	0.9	1.8	1.9
	*SCO1867*	5-Hydroxyectoine biosynthesis	0.4	−1.3	1.3	0.4
	*SCO2782*	Desferrioxamine biosynthesis	−0.6	1.4	0.7	2.7
	*SCO3218*	CDA biosynthesis	1.8	−1	3.6	0.5
	*SCO3221*	CDA biosynthesis	1.4	1.2	2.7	2.3
	*SCO5223*	Albaflavenone biosynthesis	−0.5	0.6	0.7	1.5
	*SCO5799*	Siderophore biosynthesis	−0.9	−1	0.6	0.5
	*SCO6766*	Hopene biosynthesis	0.6	−0.8	1.5	0.6
	*SCO6768*		1.4	−1.1	2.6	0.5

The abundance of the transcripts described in results is indicated; see SI Table S1 for the full transcriptome results.

^a^Transcripts not detected in the *SCO2730::Tn5062* mutant.

### Secondary metabolite transcripts with significant variations (q-value < 0.05) in the *SCO2730::Tn5062* mutant compared to wild-type strain (Wt)

**Figure 6:**
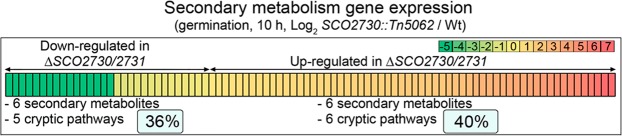
Heat map illustrating the secondary metabolism transcript abundances. Abundances correspond to log2 *SCO2730::Tn5062* / Wt. Only transcripts showing significant variations (q-value < 0.05) in the *SCO2730::Tn5062* mutant compared to the wild-type strain (Wt) are shown. *S. coelicolor* encodes 30 secondary metabolite clusters^24^: 40% up-regulated in the mutant (red colours), 36% down-regulated (green colours), 24% without variation (not shown in the figure).

We next focus on the analysis of the secondary metabolite genes up-regulated in the *SCO2730::Tn5062* mutant during germination (10 hours in sucrose-free R5A liquid cultures). The *S. coelicolor* genome encodes at least 30 predicted secondary metabolite clusters^24^. The expression of 60 genes belonging to 12 secondary metabolite clusters (40% of the total predicted)^24^ is up-regulated in the *SCO2730::Tn5062* mutant compared to the wild-type strain (Fig. 6) (Tables 1–3, SI Table S1): six well-known secondary metabolite clusters (undecylprodigiosin, hopene, geosmin, actinorhodin, 5-hydroxyectoine, CDA); four predicted cryptic pathways (lantibiotic, hexaketide, dipeptide, aromatic polyketide)^24^. *SCO3800* a gene putatively involved in secondary metabolism biosynthesis (*S. coelicolor* KEGG pathway map 01110) and *SCO1909*, a gene putatively involved in antibiotic biosynthesis (pfam03992), also were overexpressed in the mutant.

## Discussion

As introduced above, copper has pleiotropic effects in *Streptomyces* development and regulates differentiation (aerial mycelium and sporulation) and antibiotic production^13,14^. In this work we discovered additional copper effects during the vegetative stage, in germination (Fig. 1C) and growth (Fig. 1E).

**Figure 7:**
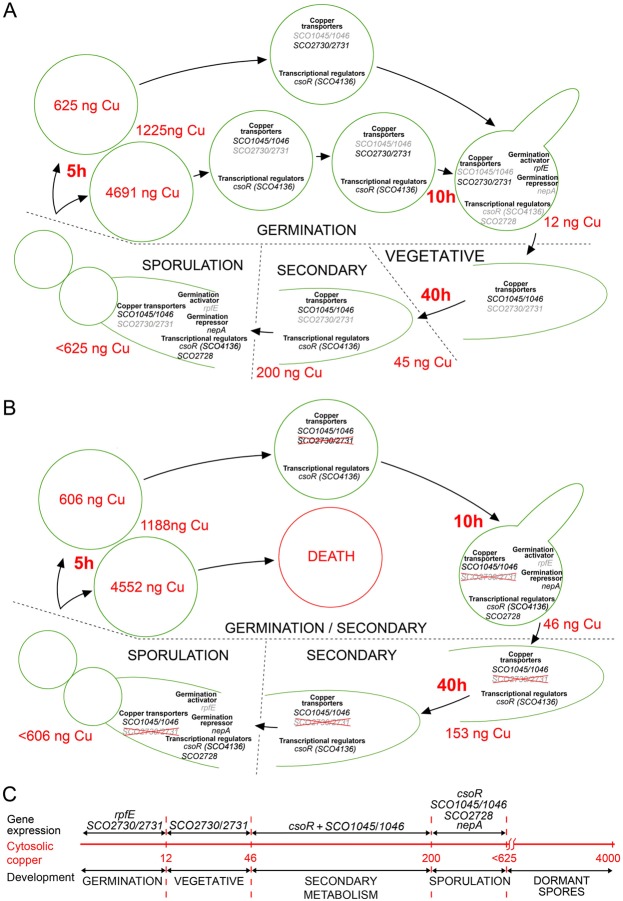
Model outlining of the effect of cytosolic copper in germination, vegetative growth, secondary metabolism and gene expression in *S. coelicolor*. **(A)** Wild-type Strain. **(B)** *SCO2730::Tn5062* mutant. **(C)** Relationship between cytosolic copper, gene expression and differentiation. Genes down-regulated are highlighted in grey.m

Based on our results, we propose the model outlined in Fig. 7, which correlates spore germination, vegetative growth, secondary metabolism and sporulation with cytosolic copper and the expression of key genes regulating these processes. In the wild-type strain (outlined in Fig. 7A), germination triggers cytosolic copper secretion thanks to the activation of the SCO2730/2731 copper chaperone/P-type ATPase (Fig. 3A,B). Cytosolic copper reaches its minimum concentration during germination (11.8 ± 0.3 ng Cu/mg protein) which suggests that the SCO2730/2731 secretion system has a higher affinity for copper than does the SCO1045/1046 chaperone/transporter that is activated at the higher cytosolic copper levels reached in the mycelium (Fig. 3A,B). This result agrees with the results of Chaplin *et al*.^17^, who demonstrated, *in vitro*, the higher copper affinity of SCO2730/2731 compared to SCO1045/1046. We postulate that the SCO1045/1046 system is also active during the first stages of copper secretion accompanying germination and until the cytosolic copper concentration reaches the low levels at which the SCO2730/2731 transporter system is activated (Fig. 3B). The low levels of cytosolic copper trigger the expression of *rpfE*, which is a key peptidoglycan lytic enzyme activated during germination^10^ (Fig. 5) (Table 1). We postulate that the asynchrony of spore germination is a consequence, at least in part, of the differences in cytosolic copper concentration in single dormant spores (Fig. 3G). The spores with the highest levels of cytosolic copper germinate more slowly than the spores with lower cytosolic copper. Cytosolic copper in wild-type single spores was below the sensitivity of the assay used in this work. However, if we assume that the variability we observed in the Wt80Cu spores (Fig. 3G) is similar to that of the Wt spores and consider an average of cytosolic copper of 1225 ng Cu/mg protein (Fig. 3D), we can expect a range of cytosolic copper in the Wt spores between 625 and 4691 ng Cu/mg of protein (Fig. 7A). During the vegetative stage of the wild-type strain, the expression of *SCO2730*/*2731* genes decreases, while the expression of the *SCO1045*/*1046* genes increases (Fig. 3B) and cytosolic copper increases (Fig. 3A). Secondary metabolism is activated (Fig. 1F,G) at cytosolic copper concentrations between 45 and 200 ng Cu/mg protein (Fig. 3A). During the sporulation stage of wild-type sporulating hyphae, we postulate the activation of a cytosolic copper accumulation mechanism that ends in high cytosolic copper levels in dormant spores (Fig. 3C). The putative zinc-responsive SCO2728 transcriptional regulator (conserved domain database accession PRK09514) might modulate the effect of cytosolic copper in sporulating hyphae. Metal sensitive transcriptional repressors can bind zinc, and also copper, which has been demonstrated, for instance, in the case of CsoR from *Bacillus subtilis*^25^. Interestingly, *SCO2728* is up-regulated 5.1-fold in the *SCO2730::Tn5062* mutant (Table 1), which perhaps indicates autoregulation of its own expression at the high copper concentrations reached in the mutant. As stated above, we postulate that sporulating hyphae accumulate copper, and the high levels of cytosolic copper activate the expression of *SCO2728*. This autoregulation is comparable to that described for the *csoR* transcriptional regulator^15^, but is at a higher copper concentration than *csoR*, because *csoR* expression is only slightly increased (1.3-fold) at the copper concentration reached in the *SCO2730::Tn5062* mutant (Table 1). Sporulating hyphae activate the expression of *nepA* (a structural cell wall protein involved in maintenance of spore dormancy)^8^, which is analogous to what happens during the germination of *SCO2730::Tn5062* mutant spores (Table 1), which maintain higher cytosolic levels than does the wild-type strain (Fig. 3D). Interestingly, *nepA* expression is not regulated by CsoR because *nepA* lacks the consensus binding sequence for CsoR^15^. The importance of copper in sporulation has already been reported^16^ and is also supported by the fact that the BCDA copper chelator represses sporulation in our SFM cultures (data not shown). The positive effect in aerial mycelium development and sporulation becomes a negative effect (delay) at high copper concentrations (over 750 µM)^15^, which was also observed in this work (Fig. 3F). We postulate that high cytosolic copper concentrations in dormant spores contribute to the inactivation of gene expression.

The acceleration in germination observed in the Wt80Cu spores (Fig. 3E) fits with the model outlined in Fig. 7A. In SFM cultures supplemented with 80 µM copper, sporulating hyphae reach the high copper levels of the dormant spores very quickly, which stops gene expression, including that of *NepA*. Consequently, the Wt80Cu spores harbour less NepA than do the Wt spores obtained in SFM non-modified cultures. The germination of the Wt80Cu spores mimics the phenotype (acceleration in germination) of the *NepA* mutant^8^. To further test this hypothesis, we analysed the germination of *NepA* mutant spores obtained in SFM cultures modified with 80 µM Cu. The copper effect in the germination of NepA mutant spores was much lower than that in the germination of the Wt80Cu spores; there was a 12% increase in germination (SI Fig. S1), compared to a 64% increase observed in Wt80Cu spores (Fig. 3E). This result corroborates that NepA has a major effect on the acceleration of spore germination mediated by copper. However, NepA is not the only effector in this phenotype, as copper still accelerates germination in the NepA mutant (Fig. 3E).

The above model also explains the phenotype of the *SCO2730::Tn5062* mutant (Fig. 7B). The absence of *SCO2730*/*2731* led to a higher copper concentration during germination (Fig. 3D). The expression of *rpfE* was not initiated (Table 1) and secondary metabolism was permanently activated (Figs 1F,G and 5). The overexpression of *SCO1046* during the germination of the *SCO2730::Tn5062* mutant spores (2.7-fold, Table 1) was unable to reduce cytosolic copper to the low levels reached in the wild-type spores (45.7 ± 8 ng Cu/mg protein in the mutant vs. 11.8 ± 0.3 ng Cu/mg protein in the wild-type strain). *SCO1045* is probably also overexpressed in the *SCO2730::Tn5062* mutant, but it has a 0.06 q-value that is over the 0.05 threshold used in this work; the abundance of this gene was not included in Table 1 (see SI Table S1). We postulate that the ungerminated dead spores that we observed (Fig. 1D), were those with the highest copper levels (4,552 ng Cu/mg protein and considering a variation in single spores comparable to the Wt80Cu spores). The absence of the *SCO2730*/*2731* copper transporter system in sporulating hyphae did not have a significant effect on the accumulation of cytosolic copper in dormant spores (Fig. 3C), which is not unexpected, considering that the main role of the *SCO2730*/*2731* copper chaperone/transporter is exerted during germination instead of sporulation (Fig. 3B).

The proposed relationship between cytosolic copper, gene expression and differentiation is summarised in Fig. 7C. Cytosolic copper concentrations under 12 ng Cu/mg protein activate the expression of *rpfE* and germination. Cytosolic copper concentrations under 45 ng Cu/mg of protein are a consequence of SCO2730/2731 copper transporter activity and correspond to vegetative growth. Cytosolic copper concentrations between 45 and 200 ng Cu/mg of protein activate the expression of CsoR and secondary metabolism and are buffered by SCO1045/1046 chaperone/transporter activity. Copper concentrations between 200 and 625 ng Cu/mg of protein modulate sporulation and activate the expression of *nepA*, *csoR* and the putative cation-responsive transcriptional regulator *SCO2728*. Copper concentrations higher than 625 ng Cu/mg protein, contribute to block gene expression in dormant spores.

The expression of *SCO2728*-*SCO2731* genes is complex and controlled by at least four promoters. Promoters 1–4 were recently identified^26^; promoter 3 was identified as being controlled by the copper dependent CsoR repressor^15^ (Fig. 1A). The four promoters affect the expression of *SCO2730*/*2731*, and complement the *SCO2730::Tn5062* mutant phenotypes (Fig. 2B–E), but they do not fully restore the wild-type phenotype (Fig. 2B–E) or the expression of the *SCO2729/2730* genes to wild-type levels (Fig. 2F,G). This result suggests the existence of further regulation of the expression of these genes, and perhaps additional uncharacterised promoters or regulatory regions in the DNA regions separating these promoters (Fig. 1A).

*SCO2731* is expressed from promoter 4 in the *SCO2730::Tn5062* mutant at a much lower amount than in the wild-type strain (Fig. 2G). Interestingly, the *SCO2730::Tn5062* complemented strain that overexpressed *SCO2731* but lacked *SCO2730* (strain 6 in Fig. 2G) showed a moderate increase in germination compared to the *SCO2730::Tn5062* mutant (Fig. 2B). This result suggests that the SCO2731 copper transporter ATPase has some transporter activity in the absence of the SCO2730 chaperone. In fact, all of the *SCO2730::Tn5062* complemented strains overexpressing *SCO2731* (strains 1, 2, 4 and 6) showed an increase in germination (Fig. 2B,G), which is probably due to the *SCO2731* copper secretion activity.

Secondary metabolites are usually defined as non-essential compounds produced at the stationary growth phase. However, this is not always the case. There are secondary metabolites that are produced during spore germination, for instance germicidin A or chalcone^27^, which both show an inhibitory effect in germination^27^. To the best of our knowledge, the *SCO2730::Tn5062* mutant is the first *Streptomyces* strain reported to enhance the expression of genes encoding some secondary metabolite pathways typical of the stationary growth phase, such as actinorhodin, undecylprodigiosin or CDA^27^, during the germination stage (10 hours, see above). Forty percent of the *S. coelicolor* predicted secondary metabolite pathways are overexpressed in the mutant, including several cryptic pathways (Fig. 6). At early time points (10 hours), most of the spores still not germinated in the *SCO2730::Tn5062* mutant (Fig. 1C), which makes the secondary metabolite enhancement observed in the mutant even more impressive, since the ungerminated inactive spores reduce the gene expression abundances (Fig. 6) and the secondary metabolite production quantified in the cultures (Fig. 1E,F).

Cations, specially Ca^2+^, but also Na^+^, K^+^, Mg^2+^, Fe^2+^, Zn^2+^, and Cu^2+^, were reported to be concentrated in dormant spores^28^. However, the variability of the concentration of these metals in single spores remains unknown. Our methodology to quantify cytosolic copper in single spores, based on a single-cell sample introduction system for triple-quadrupole inductively coupled plasma mass spectrometry (see Methods), can be adapted to quantify the concentration of these and other metals in individual spores.

Overall, in this work we discovered an unexpected pleiotropic effect of cytosolic copper that modulated germination, differentiation and secondary metabolism in *S. coelicolor*, and we propose a model correlating the copper modulated phenotypes with the expression of key copper homeostasis, and regulatory, genes. The *SCO2730/2731* genes are highly conserved in *Streptomyces*. If, as happens in *S. coelicolor*, the inactivation of these genes in other *Streptomyces* can activate or enhance 40% of the secondary metabolite pathways, this knowledge can expand the screening of new secondary metabolites from streptomycetes. It can also contribute to improving the production of already known bioactive compounds.

## Methods

### Bacterial strains and culture conditions

All *Streptomyces* and *Escherichia coli* strains used in this work are listed in Table 4. Spores were harvested from SFM solid plates^29^ after growth at 30 °C for 12 days. The differentiation analyses were carried out on sucrose-free R5A^30^ plates covered with cellophane inoculated with 10^7^ spores from a fresh water suspension and cultured at 30 °C. The samples for quantification of germination and actinorhodin and undecylprodigiosin production were obtained from 100 ml sucrose-free R5A^30^ cultures grown at 30 °C and 200 rpm in 500 ml flasks. Calcium-dependent antibiotic (CDA) production was measured on nutritive agar from Oxoid (Thermo Scientific, UK). *Escherichia coli* strains were cultured in LB and 2xTY media at 37 °C. The following antibiotics were added to select plasmid-bearing and mutant strains: ampicillin (100 μg/ml), apramycin (100 μg/ml for *E. coli*, 25 µg/ml for *S. coelicolor*), chloramphenicol (25 µg/ml), hygromycin (100 μg/ml for *E. coli*, 200 µg/ml for *S. coelicolor*), kanamycin (50 μg/ml) and nalidixic acid (25 µg/ml).

**Table 4:** Bacterial strains, plasmids and primers used in this study.

Strain	Description	Reference
*S.coelicolor* M145	SCP1^−^ SCP2^−^, reference strain.	^29^
*SCO2730::Tn5062*	*SCO2730*::*Tn5062*, Apra^R^.	This study
*E. coli* TOP10	F^-^ *mcr*A Δ(*mrr*-*hsd*RMS-*mcr*BC) φ80*lac*ZΔM15 Δ*lac*X74 *rec*A1 *ara*D139 Δ(*ara*-*leu*)7697 *gal*U *gal*K *rps*L *end*A1 *nup*G.	Invitrogen
*E. coli* ET12567	*dam-13::*Tn9*, dcm-6, hsdM*, *hsdR*.	^40^
*E. coli*		
ET12567/pUZ8002	*E. coli* ET12567 harbouring pUZ8002, a not self-transmissible plasmid which can mobilize *oriT*-containing plasmids by conjugation.	^41^
*Bacillus subtilis*	Indicator microorganism for CDA bioassay	
**Plasmids**		
pNG3	Integrative and conjugative vector, Apra^R^.	^34^
PCR™-Blunt II-TOPO®	Zero Blunt® TOPO® PCR Cloning Kit, Kan^R^.	Invitrogen
pUC57		GeneCust
**Primers**		
RT2930F	CGAGGCGACGCGGCTCATC	This study
RT2930R	ACGGCCTGTACGGAGGCGA	This study
RT3031F	GCCCGGCGCACCCATCC	This study
RT3031R	TGCCGAGCGAGACCAGCGTG	This study
RT3031F1	TCCGTCACCACCGTCTAC	This study
RT3031R1	GAGGACGGAGACCAGCAG	This study
q4758F	ATCACCGACCGGATGCCCTT	^31^
q4758R	GCCGAGCCCCGCTTCTTC	^31^
q1045F	GCATGAGCTGCGGTCACT	This study
q1045R	CCGGTGTCGTGTTGGACG	This study
q1046F	ATGACCACCAGTACGACCAG	This study
q1046R	CTTCTCCGTCGCGTAGTTGA	This study
q2730F	CACACCCGACGAGGAGTAAC	This study
q2730R	TGGAGGCGACGGCCTGTA	This study
q2731F	CGAAGTCGAGCTGCTCATC	This study
q2731R	TCTCGGTCGCGTAGTTCAC	This study
q4136F	GGATACCACAAGCAGAAGGC	This study
q4136R	AGCGCGAAGGACTGGAGG	This study
P1F	AAGATATCCTCGCTCCTGCCAGGGCG	This study
P1R	AGGCGTACGCCGTCGTTCA	This study
2730F	GTGAGTGGTCCAGGACCGGA	This study
2730R	GGCTCGAGCAGACCCGGCCGACGAGC	This study
P3.2731F	GGGATATCAGGTCGTCCTGTACGAGTG	This study
P3.2731R	AAACTAGTCTCGTGCTGTACCTGGTCG	This study
P123F	GGGATATCCATTAGGCACCCCAGGCTTT	This study
P123R	AACTCGAGTCTGGGCGGTCATGTCGTTA	This study

### DNA and RNA extraction

Genomic DNA isolation was performed following standard methods^29^. Total RNA samples were isolated as previously described^12^ using RNeasy Mini spin columns and treated with DNase I (Qiagen). The quantity and integrity of the RNA samples were measured with Nanodrop 2000 (Thermo Scientific) and 2100 Bioanalyzer (Agilent).

### Reverse transcription PCR (RT-PCR)

Co-transcription was analysed by RT-PCR. RNA obtained at 48 h sucrose-free R5A liquid cultures. Reverse transcription was performed using the SuperScript one-step RT-PCR system with Platinum Taq DNA polymerase (Invitrogen), using 200 ng of total RNA as template. Chromosomal DNA was used as template in the positive controls. RNA and the DNA polymerase included in the Platinum Taq DNA polymerase (Invitrogen) kit were used in the negative controls. RT-PCR was performed using the primers indicated in Table 4 (outlined in Fig. 1A) as follows. The first-strand complementary DNA (cDNA) synthesis was performed at 55 °C for 30 min followed by an initial denaturation at 94 °C for 2 min. Then, a touchdown was performed in 8 cycles during which the annealing temperature was reduced by 1 °C in each cycle: 94 °C for 15 s, 65 °C (Δ−1 °C) for 30 s and 68 °C for 38 s. For the next 35 cycles (94 °C for 15 s, 55 or 58 °C for 30 s and 68 °C for 38 s), the annealing temperature was set at 55 °C (for primers RT2829F/R) and 58 °C (for primers RT2930F/R, RT3031F/R and RT3031F1/R1). A final extension step was performed at 68 °C for 5 min.

### Real-Time Quantitative Reverse Transcription PCR (qRT-PCR)

A High-Capacity cDNA Reverse Transcription Kit (Applied Biosystems) was used to synthetize cDNA from 0.5 µg of RNA from two biological replicates. Real-Time PCRs were carried out on an ABI PRISM 7900 HT thermocycler (Applied Biosystems). The reactions were performed in triplicate, containing 2 µl of twofold diluted cDNA, 10 µl of SYBR Green PCR Master Mix (Applied Biosystems) and 300 nM of specific primers (listed in Table 4) in a final volume of 20 µl. *SCO4758* (amplified using primers q4758F/R)^31^ was used as a reference since its expression showed no variation between strains in our RNA-seq results (SI Table S1). The DNA contamination and primer dimer amplification were tested in negative controls replacing cDNA by RNA or water. Amplification conditions were as follows: 2 min at 50 °C, 10 min at 90 °C, 40 repetitions of 15 s at 95 °C and 1 min at 60 °C. Primer efficiencies were measured using serial dilutions of genomic DNA as template and the relative quantification of gene expression was performed by the ΔΔCt method^32^.

The average transcript fold changes and standard deviations were calculated from the two analysed biological replicates. All of the qRT-PCR quantitative data discussed in this work show large differences in the averages, which are out of the average ± SD confidence interval (i.e. error bars are not overlapping).

### *SCO2730* mutagenesis

The transposon insertion single-gene knockout library created by Professor P. Dyson’s research group^19^ was used for mutagenesis of *SCO2730*. Cosmid C46.2.D06^19^ was used to construct the *SCO2730::Tn5062* mutant strain. Gene disruption was carried out by obtaining double cross-overs via conjugation using *E. coli* ET12567/pUZ8002 as a donor strain and following the protocol described in Kieser *et al*.^29^. Mutant strains were confirmed using Southern blotting with chromosomal DNA digested with *Sal*I. Southern hybridization was carried out using established procedures with the digoxigenin-labeled 3442-bp Tn5062 *PvuII* fragment from plasmid pQM5062^33^ as a probe.

### Complementation of *SCO2730::Tn5062* mutation

The integrative plasmid pNG3^34^ was used to introduce *SCO2730* and/or *SCO2731* with different combinations of the three promoters located up-stream of the SCO2730 ORF (promoters P1-3 in Fig. 1A) into the *SCO2730::Tn5062* mutant. The specific complementation constructions cloned in pNG3 were: P_1_P_2_P_3_*SCO2730/2731*, P_1_*SCO2730/2731*, P_2_*SCO2730/2731*, P_3_*SCO2730/2731*, P_1_P_2_P_3_*SCO2730*, P_1_P_2_P_3_*SCO2731*.

The synthesis of the next genes was ordered from GeneCust Europe: *EcoR*V-P_1_P_3_*SCO2730*-*Xho*I-*Spe*I (782 bp), *Xho*I-*SCO2731*-*Spe*I (2330 bp), *EcoR*V-P_1_P_3_-*Xho*I (466 bp), *EcoR*V-P_1_P_2_P_3_*SCO2730*-*Xho*I (919 bp), *EcoR*V-P_2_*SCO2730*-*Xho*I (485 bp) (SI Table S2). These synthetic genes were provided by GeneCust cloned into pUC57 and were used to create the complementation constructs, including some intermediate forms not shown in Fig. 2.

pNG3-P_1_P_3_*SCO2730* was created as follows; pUC57-*EcoR*V-P_1_P_3_*SCO2730*- *Xho*I-*Spe*I was digested with *EcoR*V/*Spe*I. The *EcoR*V-P_1_P_3_*SCO2730*-*Xho*I-*Spe*I fragment was cloned into pNG3 digested with *EcoR*V/*Spe*I.

pNG3-P_1_P_3_*SCO2730/2731* was constructed digesting pUC57-*Xho*I-*SCO2731*-*Spe*I with *Xho*I/*Spe*I and cloning the *Xho*I-*SCO2731*-*Spe*I fragment into pNG3-P_1_P_3_*SCO2730* digested with the same enzymes.

pNG3-P_1_P_3_*SCO2731* was created digesting pUC57-*EcoR*V-P_1_P_3_-*Xho*I with *EcoR*V/*Xho*I and cloning the *EcoR*V-P_1_P_3_-*Xho*I fragment into pNG3-P_1_P_3_*SCO2730/2731* digested with the same enzymes.

pNG3-P_1_P_2_P_3_*SCO2730/2731* was created digesting pUC57-*EcoR*V-P_1_P_2_P_3_*SCO2730*-*Xho*I with *EcoR*V/*Xho*I and cloning the *EcoR*V-P_1_P_2_P_3_*SCO2730*-*Xho*I fragment into pNG3.P_1_P_3_*SCO2730/2731* digested with the same enzymes.

pNG3-P_1_*SCO2730/2731* was created as follows; P_1_ and SCO2730 were amplified by PCR from pUC57-P_1_P_3_P_2_*SCO2730* using primers P1F/R and 2730 F/R (Table 4). Fragments were amplified via PCR using Phusion High-Fidelity DNA Polymerase (Thermo), and were cloned into pCR™-Blunt II-TOPO®. The couple of DNA fragments were combined by overlap extension PCR^35^ with the primers P1F y 2730 R (Table 4). The PCR product was cloned and sequenced in pCR™-Blunt II-TOPO® using the M13 universal primers. The insert was released with *EcoR*V/*Xho*I and cloned into pNG3-P_1_P_3_*SCO2730/2731* digested with the same enzymes.

pNG3-P_2_*SCO2730/2731* was created digesting pUC57-*EcoR*V-P_2_*SCO2730*-*Xho*I with *EcoR*V/*Xho*I and cloning the *EcoR*V-P_2_*SCO2730*-*Xho*I fragment into pNG3.P_1_P_3_*SCO2730/2731* digested with the same enzymes.

pNG3-P_3_*SCO2730/2731* was created as follows; P_3_-SCO2731 was amplified by PCR from *S. coelicolor* DNA using primers P3.2731F/R. The Phusion High-Fidelity DNA Polymerase (Thermo) was used; the amplicon was cloned into pCR™-Blunt II-TOPO® and sequenced using the M13 universal primers. The insert was released with *EcoR*V/*Spe*I and cloned into pNG3 digested with the same enzymes.

pNG3-P_1_P_2_P_3_*SCO2730* was created digesting pNG3-P_1_P_2_P_3_*SCO2730.SCO2731* with *Xho*I/*Spe*I. The XhoI/*Spe*I-ends were digested with the S1 *Nuclease* (Thermo Scientific®) and the plasmid was religated.

pNG3.P_1_P_2_P_3_*SCO2731* was constructed as follows. P_1_P_2_P_3_ was amplified by PCR from pNG3-P_1_P_2_P_3_*SCO2730.SCO2731* using primers P123F/R (Table 4). The Phusion High-Fidelity DNA Polymerase (Thermo) was used; the amplicon was cloned into pCR™-Blunt II-TOPO® and sequenced using the M13 universal primers. The insert was released with *EcoR*V/*Xho*I and cloned into pNG3-P_1_P_3_*SCO2730/2731* digested with the same enzymes.

### Viability staining

Culture samples were obtained and processed for microscopy at various incubation durations, as previously described^36^. The cells were stained with propidium iodide and SYTO 9 (LIVE/DEAD Bac- Light Bacterial Viability Kit, Invitrogen, L-13152). The samples were observed under a Leica TCS-SP8 confocal laser-scanning microscope at wavelengths of 488 nm and 568 nm excitation and 530 nm (green) or 640 nm (red) emissions^36^.

### Antibiotic production and protein quantification

Undecylprodigiosin and actinorhodin were quantified spectrophotometrically, according to Tsao *et al*.^37^ and Bystrykh *et al*.^38^. For actinorhodin quantification, KOH was added to the culture samples at a final concentration of 1 N. Cellular pellets were discarded by centrifugation and actinorhodin concentration was spectrophotometrically determined at 640 nm, applying the linear Beer–Lambert relationship (*ε*_640_ = 25,320). The culture samples for undecylprodigiosin quantification were vacuum-dried, resuspended in methanol, acidified with 0.5 N HCl and spectrophotometrically assayed at 530 nm, using the Beer–Lambert relationship to estimate concentration (*ε*_530_ = 100,500).

Calcium-dependent antibiotic (CDA) production was determined via a bioassay against *Bacillus subtilis*. Oxoid nutritive agar (ONA) plates (90 mm in diameter) were inoculated with 5 µl of a *Streptomyces* spore suspension at 1 × 10^5^ spores/ml and incubated at 30 °C. After 2 days, the plates were overlaid with 5 ml of soft ONA (0.75% agar), inoculated with *B. subtilis* (OD = 0.25) and supplemented with Ca(NO_3_)_2_ (60 mM). Negative controls were performed in parallel without adding calcium. Inhibitory halos were measured after 15 h at 30 °C.

### Protein quantification

Growth was determined by measuring the protein concentration with the Bradford assay (Biorad) and a bovine serum albumin standard (Sigma). Total protein extracts were obtained mixing a volume of culture with a volume of 1 M NaOH, boiling for 5 min and removing cell debris by centrifugation at 7740 g.

### Spore germination

Germination was quantified as previously reported^8^. Briefly, germination was quantified in solid media with cellophane discs. At different developmental time points, pieces of cellophane discs were cut and processed for confocal microscopy, as described in the previous paragraph. Three biological replicates of the cultures were analysed at different developmental time points. The percentage of germination was assessed from at least 100 spores at each time point. Spores were considered to be germinating when the germ tubes were visible under the confocal microscope.

### RNA-seq and bioinformatics analysis

Next-generation sequencing (NGS) was performed by Stab Vida (Caparica, Portugal) from two biological replicates using RNA from *SCO2730::Tn5062* and *S. coelicolor* wild-type Cu-amended/non-amended spores during germination (10 h in sucrose free R5A cultures). Ribosomal RNA was depleted with the Ribo-Zero Bacteria Kit (Illumina), and the cDNA library construction was carried out using the TruSeq Stranded mRNA Library Preparation Kit (Illumina). The DNA was sequenced in the Illumina HiSeq 2500 platform using 100-bp paired-end sequencing reads (at least 20 M reads per sample). Raw data are available via the Gene Expression Omnibus database (accession GSE111126).

Bioinformatics analysis of the sequenced data was performed under the Linux operative system using the following software: FastQC to check the quality of the sequences, Cutadapt for trimming sequences, Bowtie2 for mapping with the *Streptomyces coelicolor* genome and Cuffdiff for differential expression test analysis^39^. Variation in transcript abundances was considered significant if the q-value was less than 0.05 (SI Table S1).

The SCO02730/2731 orthologues were obtained from the StrepDB (http://strepdb.streptomyces.org.uk/). SCO2730 orthologues: SLI_3079 (*S. lividans*), SAV_5332 (*S. avermitilis*), SVEN_2533 (*S*. *venezuelae*), SGR_4828 (*S. griseus*) and SCLAV_1906 (*S. clavuligerus*). SCO2731 orthologues: SLI_3080 (*S. lividans*), SAV_5331 (*S. avermitilis*), SVEN_2534 (*S*. *venezuelae*) and SGR_4827 (*S. griseus*). The *S. clavuligerus SCO2731* orthologue is not annotated in the StrepDB database, but it is located downstream of *SCLAV_1906*. Amino acid similarities were estimated using the software package Lalign (http://www.ch.embnet.org/software/LALIGN_form.html).

### Cytosolic copper quantification in dormant spores, germinated spores and mycelium

The spores or the mycelium were washed 4 times by centrifugation at 12,000 g for 10 min at 4 °C and resuspended in washing buffer (10 mM Tris-HCl pH 7.5; 1 mM EDTA). The samples were washed in washing buffer (10 mM Tris-HCl pH 7.5). For the bulk analysis of Cu in dormant spores, an acid digestion was conducted by resuspending the spores in 65% sub-boiling purified HNO_3_ at 70 °C for 1 h and then 30% H_2_O_2_ for 3 h at the same temperature. For the bulk analysis of Cu in germinated spores and mycelium, the samples were resuspended in rupture buffer (10 mM Tris-HCl pH 7.5, which is the same as the washing buffer described above). The lysis step was made using Fast-Prep (MP™ Biomedicals) with six 20-s force 6.5 cycles and with 1 minute on ice between each run (this method failed to lyse dormant spores; data not shown). Cell debris were eliminated centrifuging samples at 12,000 g for 10 min at 4 °C and discarding pellets. The resulting solutions were finally diluted with water and the total Cu content determined by ICP-MS and referred to the dry mass of the spores (1 mL spores were washed with water, dried at 100 °C to a constant weight on pre-weighted tubes) or protein (measured with the Bradford assay). Cytosolic copper in dormant spores could not be normalised against cytosolic protein, because the nitric acid treatment hydrolysed the proteins.

All the measurements were conducted in the triple quadrupole based ICP-MS Thermo iCAP-TQ (Thermo Fisher Scientific, Bremen, Germany) using the single quad mode and helium as collision gas. For bulk analysis, the ICP was equipped with a Micro Mist nebulizer, a cyclonic spray chamber (both from ESI Elemental Service & Instruments GmbH, Mainz, Germany) and an auto-sampler ASX-560 (Teledyne CETAC Technologies, Omaha, NE, USA).

All solutions were prepared using ultrapure water obtained from a Milli-Q system (Millipore, Bedford, MA, USA). HPLC-grade methanol and hydrogen peroxide for the acid digestions were both obtained from Sigma-Aldrich (Saint Louis, MO, USA). Nitric acid (65%, Suprapur quality) was purchased by Merck Millipore (Darmstad, Germany) and further purified by sub-boiling distillation. External calibrations were carried out with a Cu ICP standard CertiPur® (1000 mg.L^−1^), purchased from Merck.

### Cytosolic copper quantification in single spores

Copper was quantified in individual spores by single-cell-ICP-MS analysis. This methodology allows metal analysis in single cells by introducing diluted suspensions of spores that can be transported intact into the plasma and measured using low integration times. The arrival of several cells within the same integration time needs to be avoided by introducing highly diluted suspensions. For this aim, fresh spore suspensions (10^8^ spores spores·mL^−1^) were diluted to a final concentration of 10^5^ spores·mL^−1^ in 10% methanol to ensure the optimal nebulizer performance, according to the manufacturer and previous studies performed by our group^21^. The samples were pumped at a low flow rate of 10 μl·min^−1^ using the syringe pump SP101i (Florida, USA) fitted with a 1 mL Hamilton syringe (Nevada, USA). Samples were pumped in the triple quadrupole based ICP-MS Thermo iCAP-TQ (Thermo Fisher Scientific, Bremen, Germany) using the same parameters described above for the bulk Cu measurements. In this case, we used the microflow nebulizer EnyaMist (Burgener, Ontario, Canada) and a self-developed spray chamber, which allows a high transport efficiency of the intact spores to the plasma. Details on this experimental setup and conditions can be found in our previous work^21^. The data were recorded in the time-resolved analysis mode setting the dwell time to 1 ms and the acquisition time of each run was typically 3 min.

## Supplementary information

Supplementary Figures S1-S2 Table S2

Table S1

## Data Availability

RNA-seq raw data are available via the Gene Expression Omnibus database (accession GSE111126). The datasets generated during the current study are available from the corresponding author on request.
