# Supplementary material for: Cytosolic copper is a major modulator of germination, development and secondary metabolism in Streptomyces coelicolor
Source: Sci Rep. 2019 Mar 12;9:4214. doi: 10.1038/s41598-019-40876-0 (PMC6414726; doi:10.1038/s41598-019-40876-0)
Supplement: Supplementary file 1 — Supplementary Figures S1-S2 Table S2 [file 41598_2019_40876_MOESM1_ESM.pdf]

Cytosolic copper is a major modulator of germination, development and secondary metabolism in *Streptomyces coelicolor*

Nathaly González-Quiñónez[1], Mario Corte-Rodríguez[2], Roberto Álvarez-Fernández-García[2], Beatriz Rioseras[1], María Teresa López-García[1], Gemma Fernández-García[1], María Montes-Bayón[2], Angel Manteca[1a*] and Paula Yagüe[1a]

[1] Área de Microbiología, Departamento de Biología Funcional, IUOPA and ISPA, Facultad de Medicina, Universidad de Oviedo, 33006 Oviedo, Spain.

[2] Department of Physical and Analytical Chemistry, Faculty of Chemistry and ISPA, Universidad de Oviedo, 33006 Oviedo, Spain.

Figure S1. Copper effect in the *S. coelicolor* Δ*nepA* mutant. **(A)** Percentage of germination (three biological replicates) in the Δ*nepA* and the Δ*nepA*Cu in sucrose free R5A medium (6 hours). **(B)** Macroscopic view of sporulation (grey colour) of the Δ*nepA* strain in SFM and SFM amended with 80 μM $CuSO_4$. Δ*nepA*, *nepA* mutant. Δ*nepA*Cu, spores of the *nepA* mutant obtained in 80 μM $CuSO_4$ amended SFM cultures.

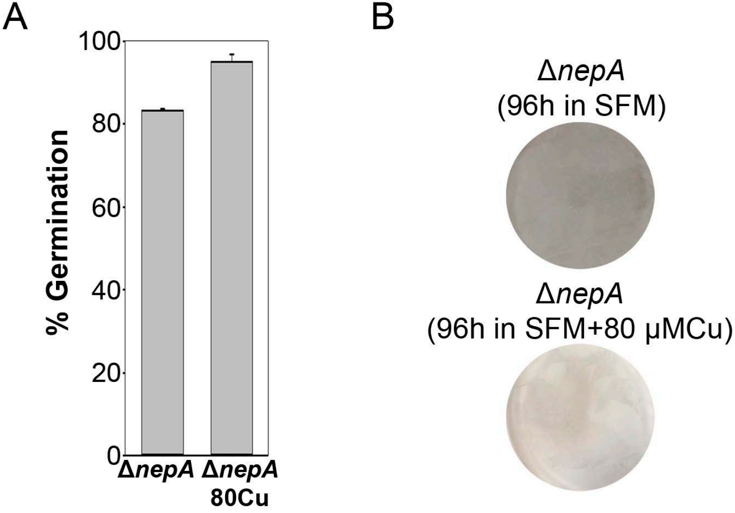

Figure S2. Full-length gels used to create Figure 1B

1 - 420 bp

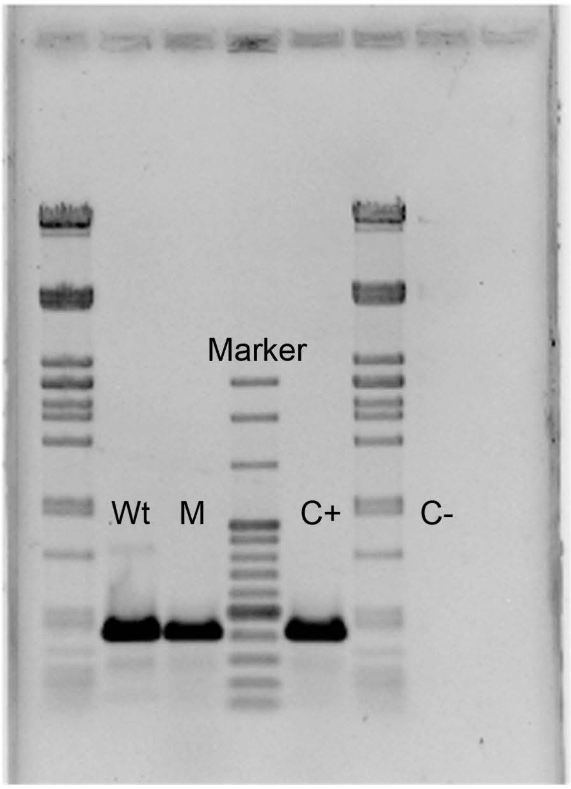

2 - 480 bp

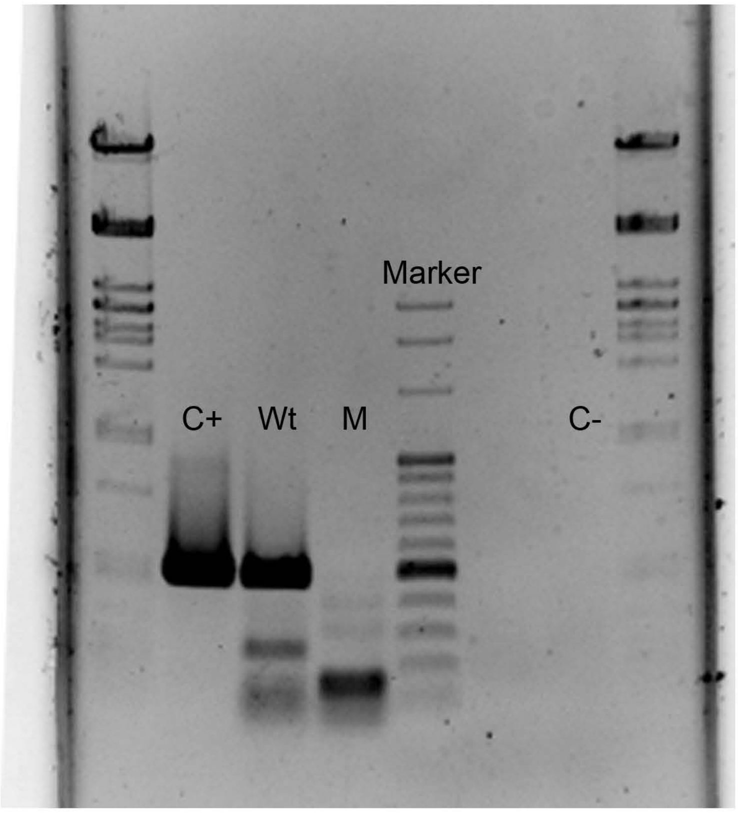

3 - 594 bp

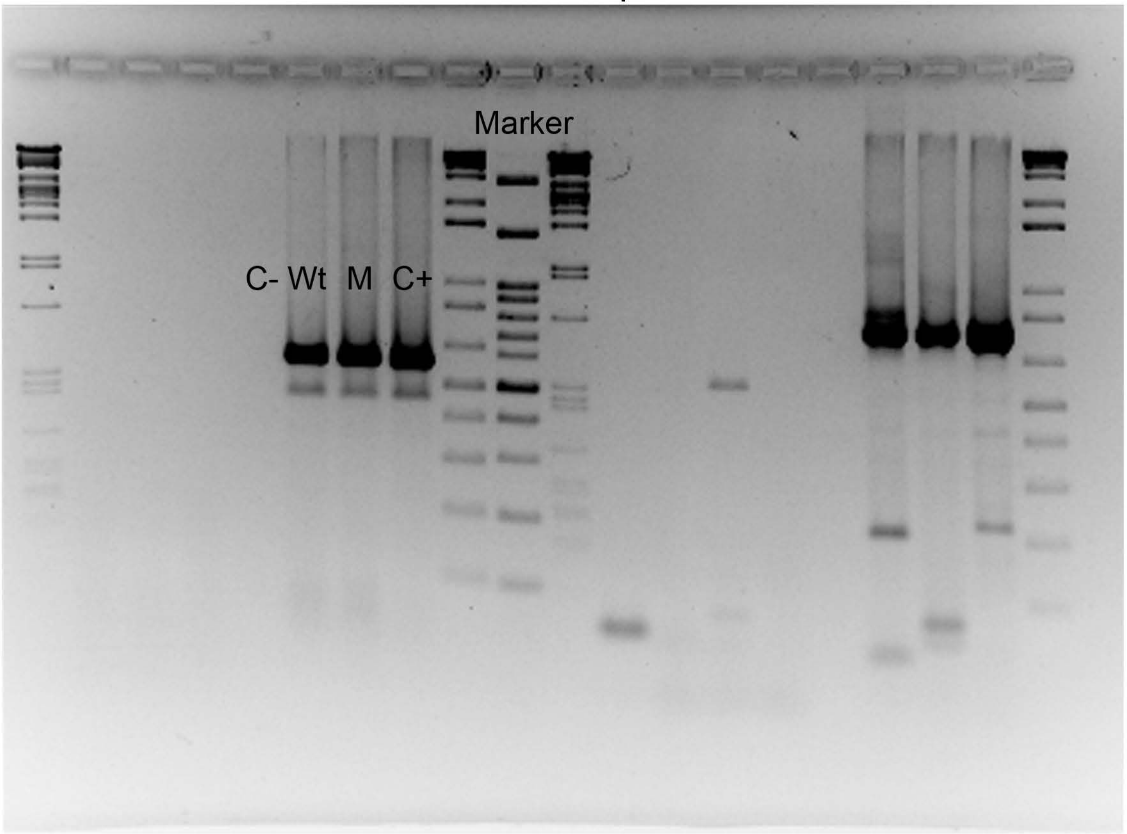

Figure S2. Full-length gels used to create Figure 1B.

4 - 626 bp

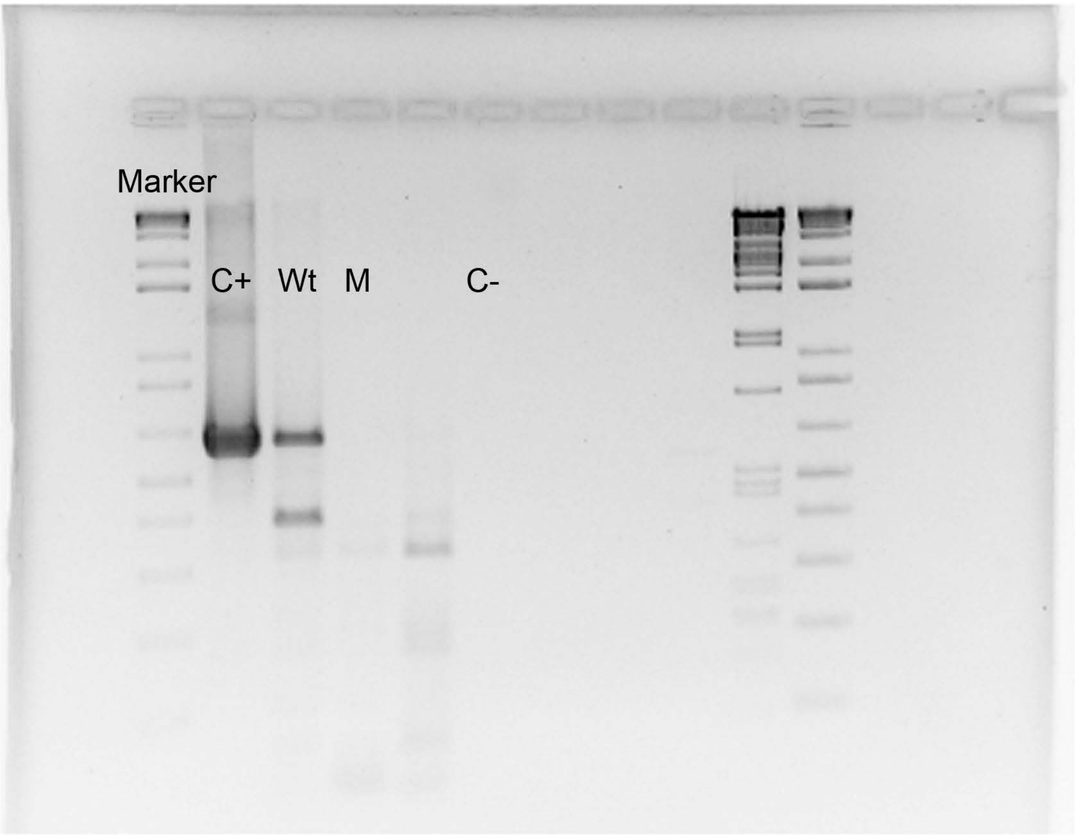

Table S2. Synthetic sequences. P_1-P_3 are gene promoters (see Fig. 1A). Restriction enzyme cutting sequences are in red; P_1 in green; P_2 in blue; P_3 in brown; *SCO2730/2731* ORFs in black. A sequence upstream of the *SCO2730/2731* ORFs, long enough to include the RBS, but not P_3 or P_4, was included (highlighted in yellow).

EcoRV-P_1P_3RBS ***SCO2730-XhoI*-TTTT-*SpeI*** **782 bp**	***EcoRV***-$P_1P_2P_3$RBS ***SCO2730-XhoI*** **919 bp**
GATATCCTCGCTCCTGCCAGGGCGAGGCCGCCTTCCCGCGCCGGAGGCGT<br>TCGGTGGGGGAACCGGCGCCCCGGCCGTGTGCCGTCGGGTCGTGCGCCAT<br>CGCGTTCCCTCCTTGCCCGCGCCCTTGCGTCACCCCGGTTTCTAGCCCCG<br>TGCGTGCGGCCTGTCAATGTTTTGTCCGGACATTCGGACGACGTAGTCCG<br>AGTGGCGGTGGAGGGGCGTACGGCGGAACACCTCCGTCCGCCGCAGTCAA<br>CGCGCCGCGCCTGAACGACGGCGTACGCCTTACGAGAAGGCCGGTTTCAT<br>GGTGGAGGGGGTGCGCCGGGAGGCCGCCCTGCGGGGCGGCGCGTGGGTGG<br>ACGGCGTACTGATGGCGGTCCTCGACCACGAGTGGGAGGCGCGGGCACGC<br>ACGGACGCGACGCAGGTGACGGGTGAGCCTCGCCTTTATACCCCCTAGGG<br>GTAAGGTGGGGTGAGTGGTCCAGGACCGGACCACGGGTACCCGCCCTGTG<br>CACACCCGACGAGGAGTAACGACATGACCGCCCAGACCGACACCACCCAG<br>GACTCCGTCACCACCGTCTACAAGGTGAGCGGAATGAGCTGCGGCCACTG<br>CGAGGGCGCCGTCTCCGGCGAGATCTCCGAGATCGCGGGCGTCGCCTCCG<br>TACAGGCCGTCGCCTCCACCGGCGAGGTCACCGTCGTCTCCGGGGCACCC<br>CTGGACGACGCCGCCGTGCGCGCCGCCGTCGACGAGGCGGGCTTCGAGCT<br>CGTCGGCCGGGTCTGACTCGAGTTTTACTAGT	GATATCCTCGCTCCTGCCAGGGCGAGGCCGCCTTCCCGCGCCGGAGGCGTTCGGTGGGGGA<br>ACCGGCGCCCCGGCCGTGTGCCGTCGGGTCGTGCGCCATCGCGTTCCCTCCTTGCCCGCGC<br>CCTTGCGTCACCCCGGTTTCTAGCCCCGTGCGTGCGGCCTGTCAATGTTTTGTCCGGACAT<br>TCGGACGACGTAGTCCGAGTGGCGGTGGAGGGGCGTACGGCGGAACACCTCCGTCCGCCGC<br>AGTCAACGCGCCGCGCCTGAACGACGGCGTACGCCTGCCTGGGTGGCGTCCCGCCCCCGCA<br>ACCGCAACCGGTAGCGACGGACGACGGAGGGGCGCAGGAGCGGAGGGGCGGAGGGGCGGA<br>GGGAAAACCGTTCAGTGCCCCACCGCCGGGTGGGGCACAGTGCCGTGCATGGACTTCTCCG<br>TACGAGAAGGCCGGTTTCATGGTGGAGGGGGTGCGCCGGGAGGCCGCCCTGCGGGGCGGCG<br>CGTGGGTGGACGGCGTACTGATGGCGGTCCTCGACCACGAGTGGGAGGCGCGGGCACGCAC<br>GGACGCGACGCAGGTGACGGGTGAGCCTCGCCTTTATACCCCCTAGGGGTAAGGTGGGGTG<br>AGTGGTCCAGGACCGGACCACGGGTACCCGCCCTGTGCACACCCGACGAGGAGTAACGACA<br>TGACCGCCCAGACCGACACCACCCAGGACTCCGTCACCACCGTCTACAAGGTGAGCGGAAT<br>GAGCTGCGGCCACTGCGAGGGCGCCGTCTCCGGCGAGATCTCCGAGATCGCGGGCGTCGCC<br>TCCGTACAGGCCGTCGCCTCCACCGGCGAGGTCACCGTCGTCTCCGGGGCACCCCTGGACG<br>ACGCCGCCGTGCGCGCCGCCGTCGACGAGGCGGGCTTCGAGCTCGTCGGCCGGGTCTGACT<br>CGAG
EcoRV-P_1P_3 RBS -***XhoI*** **466 pb**	***EcoRV***-P_2 RBS ***SCO2730-XhoI*** **485 pb**
GATATCCTCGCTCCTGCCAGGGCGAGGCCGCCTTCCCGCGCCGGAGGCGT<br>TCGGTGGGGGAACCGGCGCCCCGGCCGTGTGCCGTCGGGTCGTGCGCCAT<br>CGCGTTCCCTCCTTGCCCGCGCCCTTGCGTCACCCCGGTTTCTAGCCCCG<br>TGCGTGCGGCCTGTCAATGTTTTGTCCGGACATTCGGACGACGTAGTCCG<br>AGTGGCGGTGGAGGGGCGTACGGCGGAACACCTCCGTCCGCCGCAGTCAA<br>CGCGCCGCGCCTGAACGACGGCGTACGCCTTACGAGAAGGCCGGTTTCAT<br>GGTGGAGGGGGTGCGCCGGGAGGCCGCCCTGCGGGGCGGCGCGTGGGTGG<br>ACGGCGTACTGATGGCGGTCCTCGACCACGAGTGGGAGGCGCGGGCACGC<br>ACGGACGCGACGCAGGTGACGGGTGAGCCTCGCCTTTATACCCCCTAGGG<br>GTAAGGTGGGCTCGAG	GATATCGCCTGGGTGGCGTCCCGCCCCCGCAACCGCAACCGGTAGCGACGGACGACGGAG<br>GGGCGCAGGAGCGGAGGGGCGGAGGGGCGGAGGGAAAACCGTTCAGTGCCCCACCGCCGGG<br>TGGGGCACAGTGCCGTGCATGGACTTCTCCGCCCCCTAGGGGTAAGGTGGGGTGAGTGGTC<br>CAGGACCGGACCACGGGTACCCGCCCTGTGCACACCCGACGAGGAGTAACGACATGACCGC<br>CCAGACCGACACCACCCAGGACTCCGTCACCACCGTCTACAAGGTGAGCGGAATGAGCTGC<br>GGCCACTGCGAGGGCGCCGTCTCCGGCGAGATCTCCGAGATCGCGGGCGTCGCCTCCGTAC<br>AGGCCGTCGCCTCCACCGGCGAGGTCACCGTCGTCTCCGGGGCACCCCTGGACGACGCCGC<br>CGTGCGCGCCGCCGTCGACGAGGCGGGCTTCGAGCTCGTCGGCCGGGTCTGACTCGAG

_Xho_I- RBS _SCO2731_-_Spe_I **2330 pb**

CTCGAGGCCCGGCGCACCCATCCCGTTCTGGAGTCCGGACCATGACCAGCACCACCGCGGACACGCGCACCTCCACGACGGCCACCGGGCCCGATCCCGGCGTCGCCGAAGTCG
AGCTGCTCATCGGCGGGATGACCTGCGCCTCCTGCGCGGCCCGCGTCGAGAAGAAGCTCAACCGCATGGACGGTGTCACCGCCACGGTGAACTACGCGACCGAGAAGGCCCGGG
TCAGCTACCCGGCGACCACCGGGGTCGCCGACCTGATCGCCACCGTCGTGAAGACCGGGTACACGGCCGAGGAGCCCGCGCCGCCACCGGAGCCCGCCGACGAGGCCGGGGCCG
GGGAGCGCGGCACCGGGGACGGCGGGAGCGACCCGGAACTGTCGGCCCTGCGTCAGCGCCTGCTGGTCTCCGTCCTCCTCGCCGCCCCCGTCGTGCTGCTCGCGATGGTCCCGG
CGCTCCAGTTCGACAACTGGCAGTGGCTCTCGCTCACTCTGGCCGCGCCCGTCGTGGTCTGGGGCGGGCTGCCCTTCCACCGCGCCGCCTGGACCGGCCTGCGGCACGGCGCGG
CCACCATGGACACGCTGGTCTCGCTCGGCACGCTGGCGGCGTTCGGCTGGTCCCTGTGGGCGCTGTTCTTCGGGGACGCGGGCATGCCGGGCATGCGGCACGGCTTCGACCTCA
CCGTCTCCCGCACCGACGGCACCTCCGCGATCTACCTGGAGGCCGCCGCCGGGGTCACCGCCTTCCTGCTCCTGGGCCGCTGGCTGGAGGCCCGCTCCAAGCGCCGCGCGGGGG
CCGCCCTGCGGGCGCTGATGGAGCTGGGCGCCAAGGACGTGGCCGTACTGCGGGCCGGGCGCGAGGTGCGGATACCGGTGGCCCGCCTGGCGGTGGGCGACCGGTTCGTCGTAC
GCCCCGGCGAGAAGATCGCCACCGACGGCACGGTGGCCGAGGGCGCCTCGGCCGTGGACGCCTCGCTGCTGACCGGCGAGTCCGTGCCGGTGGACGTCGCCGTCGGCGACACCG
TCACCGGCGCCACGGTCAACGCCGGGGGCCGGCTGGTGGTCGAGGCGACCCGGGTGGGCGCGGACACGCAGCTGGCGCGGATGGCGAAGCTGGTGGAGGACGCGCAGAGCGGCA
AGGCGCAGGTGCAGCGGCTCGCCGACCGGATCTCGGGGATCTTCGTCCCCGTCGTGCTGCTGATCGCGTTCGCCACCTTCGGCGGCTGGCTCGGTGCCACCGGTGACACGGTCG
CCGCGTTCACCGCGGCCGTCGCCGTCCTGATCATCGCCTGCCCGTGCGCGCTGGGCCTGGCCACCCCGACGGCGCTGCTGGTCGGCACCGGTCGCGGCGCCCAGCTCGGCATCC
TCATCAAGGGCCCGGAGGTACTGGAGAACACGCGCCGCGTCGATACGGTCGTCCTGGACAAGACCGGCACCGTCACCACCGGCCGCATGACCCTGCACGAGGTGTACGCCGCCG
AGGGCACCGACGAGGAGGAGCTGCTGCGGCTCGCGGGCGCCGTCGAGCACGCCTCCGAGCACCCGGTGGCCCGCGCGATCGCCGCAGGCGCCGAGGCACGGCTCGGCACGCTGC
CGGGCGCCGAGGACTTCGAGAACGTCCCCGGGCGCGGCGCACGCGCCCGCGTGGCGGGCCACGAGGTGGCCGTGGGGCGCCTCCACGACACCCTGCCGCCCGAGGTGTCCCGGG
CCAGGGACGAGGCCGAGCAGCGGGGCCGTACGGCCGTCGTCGTCGGCTGGGACGGCGCGGCGCGCGGGGTGCTCGCCGTGGCGGACGCGGTCAAGGAGACCAGCGCCGAGGCGG
TGGCCGGGCTGCGCCGGCTGGGTCTGACCCCGGTCCTGCTGACCGGCGACAACCGCCGGGTGGCCGAGTCGGTCGCGGCGGCCGTCGGCATCGACGAGGTGATCGCCGAGGTGC
TGCCCGAGGACAAGGTCGCGGTGGTGGAGCGGCTGCGGGCCGAGGGCCGTACGGTCGCCATGGTCGGCGACGGCGTCAACGACGCGGCCGCCCTCGCCACCGCCGATCTGGGCC
TGGCGATGGGTACGGGGACGGACGCGGCGATCGAGGCGGGCGACCTGACGCTGGTGCGCGGTGACCTGCGGGTGGCGGCGGACGCGATCCGGCTCTCCCGGCGCACCCTGGCCA
CGATCAAGGGCAATCTCGTGTGGGCCTTCGGCTACAACGTGGCCGCGCTGCCGCTGGCCGCCGCCGGGCTGCTGAACCCGATGATCGCGGGGGCGGCGATGGCCTTCTCCTCGG
TCTTCGTGGTCACCAACAGCCTTCGGCTGCGCGCTTTCCGATAGACTAGT
